# A Critical Review on Emerging Trends in Dry Powder Inhaler Formulation for the Treatment of Pulmonary Aspergillosis

**DOI:** 10.3390/pharmaceutics12121161

**Published:** 2020-11-28

**Authors:** Shen Nam Cheng, Zhi Guang Tan, Manisha Pandey, Teerapol Srichana, Mallikarjuna Rao Pichika, Bapi Gorain, Hira Choudhury

**Affiliations:** 1 School of Pharmacy, International Medical University, Kuala Lumpur 57000, Malaysia; cheng.shennam@student.imu.edu.my (S.N.C.); tan.zhiguang@student.imu.edu.my (Z.G.T.); 2 Department of Pharmaceutical Technology, School of Pharmacy, International Medical University, Jalan Jalil Perkasa, Bukit Jalil, Kuala Lumpur 57000, Malaysia; 3 Drug Delivery System Excellence Center, Prince of Songkla University, Songkhla 90110, Thailand; teerapol.s@psu.ac.th; 4 Department of Pharmaceutical Technology, Faculty of Pharmaceutical Sciences, Prince of Songkla University, Songkhla 90110, Thailand; 5 Centre for Bioactive Molecules and Drug Delivery, Institute for Research, Development and Innovation (IRDI), International Medical University, Kuala Lumpur 57000, Malaysia; mallikarjunarao_pichika@mu.edu.my; 6 Department of Pharmaceutical Chemistry, School of Pharmacy, International Medical University, Kuala Lumpur 57000, Malaysia; 7 School of Pharmacy, Faculty of Health and Medical Sciences, Taylor’s University, Subang Jaya, Selangor 47500, Malaysia; bapi.gorain@taylors.edu.my; 8 Centre for Drug Delivery and Molecular Pharmacology, Faculty of Health and Medical Sciences, Taylor’s University, Subang Jaya, Selangor 47500, Malaysia

**Keywords:** pulmonary aspergillosis, pulmonary delivery, dry powder inhalers, nebulizer, antifungal agents, nanotechnology, nanocarriers

## Abstract

Pulmonary aspergillosis (PA), a pulmonary fungal infection caused by *Aspergillus* spp., is a concern for immunocompromised populations. Despite substantial research efforts, conventional treatments of PA using antifungal agents are associated with limitations such as excessive systemic exposure, serious side effects and limited availability of the therapeutics in the lungs for an adequate duration. To overcome the limitations associated with the conventional regimens, pulmonary delivery of antifungal agents has become a focal point of research because of the superiority of local and targeted drug delivery. Dry powder inhalers and nebulized formulations of antifungal agents have been developed and evaluated for their capability to effectively deliver antifungal agents to the lungs. Moreover, progress in nanotechnology and the utilization of nanocarriers in the development of pulmonary delivery formulations has allowed further augmentation of treatment capability and efficiency. Thus, the following review provides an insight into the advantages and therapeutic potential of the utilization of nanocarriers in pulmonary delivery of antifungal agents for the treatment of PA. In addition, discussions on formulation aspects and safety concerns together with the clinical and regulatory aspects of the formulations are presented, which suggest the possibility and desirability of utilization of nanocarriers in the treatment of PA.

## 1. Introduction

**Figure 1:**
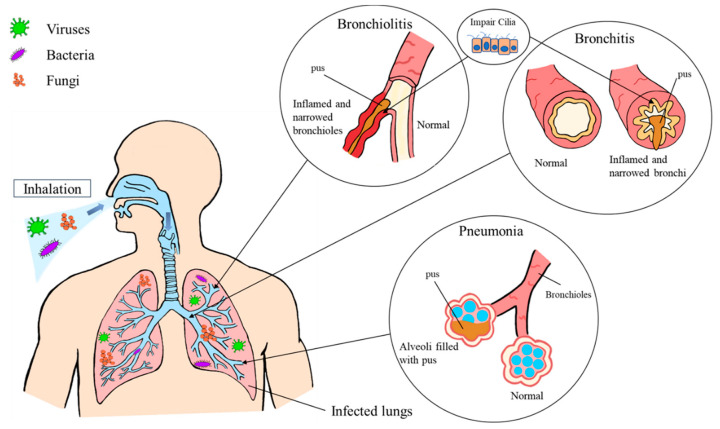
Representation of different types of lung infections.

Then lungs are one of the most common sites that can be infected by a variety of microorganisms such as bacteria, viruses, parasites and fungi. Lung infections contribute to high mortality and morbidity among immunocompromised patients [1]. There are several types of lung infections including bronchitis, bronchiolitis, and pneumonia (Figure 1). Infectious bronchitis is the inflammation of the airways and bronchi in which most of the cases involve viral infections. Patients with bronchitis usually present with signs and symptoms such as cough, fever, headache, nasal congestion, and dyspnea. In the management of bronchitis, the mainstay treatments involve symptom management and supportive care [2]. Bronchiolitis, on the other hand, is the inflammation of the smaller airways and bronchioles in the lungs. Acute bronchiolitis is more common in the pediatric population [3]. Adults are mostly affected by respiratory bronchiolitis mainly related to smoking [4]. The most common form of bronchiolitis is infectious bronchiolitis, which can be further classified into acute or chronic infectious bronchiolitis. Acute bronchiolitis is normally caused by infection by viruses such as parainfluenza virus and respiratory syncytial virus or bacteria including *Mycoplasma pneumoniae* and *Staphylococcus aureus*, while chronic infectious bronchiolitis is mostly caused by both bacteria and mycobacteria [5]. Bronchiolitis shares common signs and symptoms with bronchitis. Due to the lack of curative treatment, the management of bronchiolitis is mainly supportive by maintaining hydration and oxygenation [3].

Among the types of lung infections, pneumonia is the most common and widely known lung infection that is widespread, recently causing a high burden on health. It is also the top leading infection that results in the death of children below five years old in developing countries [6]. Pneumonia results in the inflammation of the alveoli or air sacs in one or both lungs causing them to fill up with pus. Generally, patients with pneumonia present with symptoms ranging from mild to severe including fever, confusion, chest pain, cough, dyspnea, and sputum production. Pneumonia can be further divided into different categories in different manners based on the causative microorganisms or the location of the patients when infected. Viruses, bacteria and fungi are the main causative microorganisms that cause pneumonia through the bloodstream or inhalation of the microorganisms. Viruses that commonly infect the lungs and lead to pneumonia are influenza virus, parainfluenza, respiratory syncytial virus, rhinovirus, and adenovirus [7]. Apart from that, coronavirus, an enveloped single-stranded RNA virus normally found in mammals and birds also can also cause pneumonia [8]. Novel coronavirus disease (COVID-19) caused by severe acute respiratory syndrome (SARS)-coronavirus (CoV)-2 which belong to a member of Coronaviridae, has caused respiratory failure in a huge number of patients [9,10].

Bacterial infections of the lungs are a common complication of viral infections as the viruses damage the lungs’ defense system and make the lungs vulnerable to other infections [6]. Both Gram-positive and Gram-negative bacteria are involved in lung infections and known to cause pneumonia. For example, *Streptococcus pneumoniae, Hemophilus influenzae, Staphylococcus aureus,* and *Pseudomonas* spp. are causatives of such infections [11]. In the treatment of pneumonia, antimicrobials administered via oral, inhalational and intravenous delivery routes are provided to the patients based on the infection severity, while the choice of antimicrobials used depends on the microorganisms that have infected the patient [12].

In short, a wide range of microorganisms can infect the lungs. However, fungal infections in the lungs are more common, severe and critical, particularly in immunocompromised patients. Therefore, the increase of patients with an impaired immune system due to suffering from immunodeficiency disorders, receiving immunosuppressive drugs or undergoing chemotherapy, increases the possibilities of fungal infections, which represent a serious threat to public health [13]. This review is focused on the utilization of nanocarriers in pulmonary delivery for effective and safe treatment of pulmonary fungal infections, with special emphasis on PA.

## 2. Pulmonary Fungal Infection

There are more than 150 million people are suffering from serious fungal infections and the mortality associated with fungal infections is more than 1.6 million. The incidence and prevalence of fungal disease across the globe varies according to the socio-economic status and geo-ecological characteristics of each nation. Populations who are non-immunocompetent or with impaired lung function such as patients who suffer from HIV/AIDS, tuberculosis (TB), chronic obstructive pulmonary disease, asthma and cancers are at-high risk of fungal infection [14]. Further, the preliminary cause of pulmonary fungal infections consists of endemic fungi such as *Histoplasma* spp., *Coccidioides* spp., *Blastomyces dermatitidis*, *Paracoccidioides brasiliensis, Aspergillus* spp., *Crytococcus* spp., responsible for the common types of pulmonary fungal infection, such as histoplasmosis, coccidioidomycosis, blastomycosis, paracoccidioidomycosis, aspergillosis and cryptococcosis, respectively [15,16]. These fungal infections are generally acquired through the inhalation of airborne fungal spores [17]. When the fungi spores are inhaled in a substantial amount, the innate defenses of the individuals will be overwhelmed, especially in patients who are non-immunocompetent. Colonization of the fungi spores in the respiratory tract will then occur either by a direct allergenic pathway or indirect colonization through contact of the pathogenic fungi with cell wall constituents and proteases. This will lead to the manifestation of the disease, which includes the respiratory and constitutional symptoms such as dyspnea, fatigue, cough and even hemoptysis [16]. Generally, antifungal agents are used as first-line therapy to treat infections and abate the symptoms [16]. Itraconazole (ITZ) is generally recommended as the first-line therapy for mild to moderate pulmonary fungal infections. Patients with severe pulmonary fungal infections should be treated with liposomal amphotericin B (AmB) until clinical improvement followed by ITZ consolidation therapy, whereas patients with severe coccidioidomycosis should be treated with high dose fluconazole. Depending on the specific causative agent, the severity of the illness, and the immune status of the patient, the duration of treatment for pulmonary fungal infection can range from 6 weeks to more than 12 months [17]. For instance, patients with histoplasmosis need to be treated with antifungal agents for six to twelve weeks, whereas patients with paracoccidioidomycosis, blastomycosis and aspergillosis need to be treated with antifungal for at least 6 months [18]. Among the fungal infections, aspergillosis is a more prevalent fungal infection that requires longer duration antifungal treatment compared to other types of fungal infections (<1 million cases annually). The mortality rate of chronic pulmonary aspergillosis (PA) is around 40%, even with long-term antifungal therapy. More details about PA are discussed in the following section.

### Pulmonary Aspergillosis

*Aspergillus* species are ubiquitous in the environment and the most common opportunistic type of fungi that cause pulmonary infection [19]. More than 14 million people are affected by PA worldwide, with syndromes that are immensely diverse depending on the immune status of the patients. In descending order of frequency, the five most clinically relevant fungi responsible for PA in humans are: *Aspergillus fumigatus*, *A. flavus*, *A. niger*, *A. terreus,* and *A. nidulans*. Allergic bronchopulmonary aspergillosis (ABPA), invasive PA (IPA) and chronic PA (CPA) are the three major *Aspergillus*-related bronchopulmonary syndromes responsible for the most morbidity and mortality worldwide, with ABPA (>4 million cases annually) and CPA (~3 million cases annually) being more prevalent than IPA (>300,000 cases annually). Out of approximately 3 million cases of CPA, around 1.2 million cases are estimated to be a sequel of previously treated pulmonary TB [20,21]. The predisposing factors for PA include weakened host immune integrity, pre-existing pulmonary disease and impaired bronchial defense mechanisms [15].

**Figure 2:**
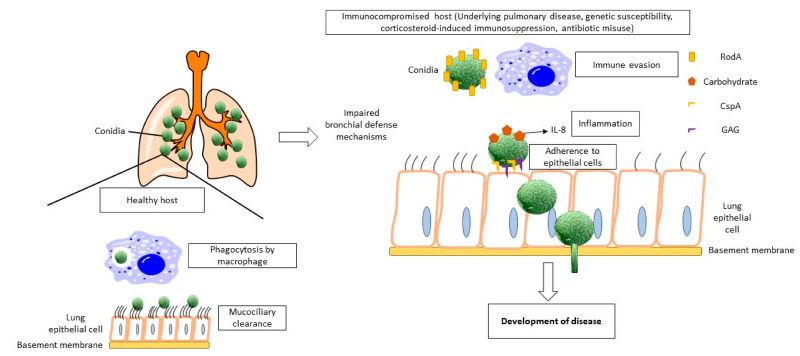
Pathophysiology of pulmonary aspergillosis.

One can become infected by *Aspergillus* spp. when conidia released into the atmosphere are inhaled and deposited in the respiratory tract. These small diameter (3–5 µm) conidia are usually cleared by the bronchial and innate defense mechanisms of the immunocompetent hosts without underlying pulmonary disease [15,20]. However, through inevitable repeated respiratory exposure to the conidia, these conidia will eventually penetrate into the alveolar spaces resulting in saprophytic colonization in the lung cavities and damaging the surrounding parenchyma. Colonization of the conidia is promoted by some *Aspergillus* factors, which include the surface protein RodA, carbohydrates, GPI-anchored protein CspA, exopolysaccharide galactosaminogalactan and extracellular proteases. These features allow *Aspergillus* spp. to persist in the airways while evading destruction by the immune system (Figure 2). On the other hand, host factors that promote *Aspergillus* colonization include underlying immune response abnormalities, pulmonary disease, genetic susceptibility, antibiotic misuse, and corticosteroid treatment [21,22]. Fungal colonization will ultimately lead to the development of the disease, which manifests as a localized inflammation response, pleural thickening, parenchymal fibrosis, expansion of the colonized cavities with or without the formation of aspergilloma, the fungal growth detached from the cavity wall [20].

For the treatment of PA, surgical resection is recommended for patients with simple aspergilloma or with non-progressive localized CPA with no significant comorbidities, whereas antifungal treatment is recommended for symptomatic patients. The first-line therapy for PA is ITZ (200 mg b.i.d., ≥6 months) while the second-line therapy is voriconazole (VRZ) (150–200 mg b.i.d., ≥6 months) where both drugs require therapeutic drug level monitoring. The third-line therapy is posaconazole (PCZ) (400 mg b.i.d., ≥6 months) or isavuconazole (loading dose: 200 mg t.i.d. on day 1 + 2 followed by 200 mg q.d. maintenance), whereas the fourth-line therapy is intravenous drugs, which are AmB (0.7–1.0 mg/kg/day), caspofungin (50–70 mg q.d.) and micafungin (150 mg q.d.). The third-line therapy is used in case of *Aspergillus* strain resistance and untolerated side effects to ITZ and VRZ. Due to the inhibition of CYP3A4 by all triazoles, there are high possibility of drug-drug interactions. The fourth-line therapy is used as the last resort when there are signs of triazole toxicities, drug interaction problems, clinical failure, and panazole resistance. However, echinocandins are preferred due to significant nephrotoxicity associated with the long-term use of intravenous AmB [18,20]. The number of classes of antifungal drugs is limited and are associated with some treatment limitations. For instance, current triazole formulations include capsules and oral suspensions but these oral formulations are associated with tolerability and safety issues due to erratic absorption, poor lung distribution of the drug, gastrointestinal side effects, high inter-individual pharmacokinetic variability and extensive drug-drug interactions [23,24]. Besides, administration of antifungal agents through oral and intravenous routes is known to result in high systemic exposure of the drug, which increases the risk of systemic toxicity [25,26]. Hence, the administration of triazoles via other routes has been studied to discover advanced drug delivery systems that have lesser limitations than conventional formulations and capable of targeted drug delivery.

## 3. Novel Approaches for Lung Drug Delivery against Fungal Infection

Novel drug delivery approaches have been widely investigated for the last two decades to overcome the limitations of the conventional formulations of antifungal drug for lung infections, such as high doses, frequent dosing, side effects and systemic toxicity. Recently, nanocarriers have gained increased attention for their capability and usage in drug delivery. The possibility of tuning the physicochemical properties of nanocarriers to obtain desired formulation properties makes them promising drug carriers with minimal side effects [27,28,29]. Besides the incorporation of antifungal agents into nanocarriers, these could also provide drug property improvements such as better drug pharmacokinetics profiles, penetration through tissues, aqueous solubility, drug efficacy, drug stability and reduced side effects [30].

### 3.1. Pulmonary Delivery of Antifungal Agents

Pulmonary fungal infections are difficult to treat as the fungal spores penetrate deep into the small airways and conventional routes of administration may not achieve delivery of an optimum level of antifungal at the site of infection due to poor solubility, permeability and rapid clearance of the antifungal. Hence, pulmonary route of administration for antifungal agents have been ventured into due to their advantages of targeted delivery of drug to the deep lung, reduction of systemic drug exposure and less dosing frequency as the drug administered through this route can bypass the first-pass effect. Particularly, the use of nanocarriers in delivering antifungal agents through the pulmonary route has been investigated due to their ability to improve the stability and bioavailability of antifungal agents. Antifungal agents encapsulated in nanocarriers can be delivered to the lungs through dry powder inhalers (DPI) in the form of dry powders and nebulizers in the form of solutions or suspensions [31]. More details on DPI and nebulized formulations will be discussed in the next section.

#### 3.1.1. Dry Powder Inhaler Formulations

Pulmonary drug delivery is an effective way of targeting pulmonary fungal infections. Local drug action can be achieved through pulmonary drug delivery of dry powder formulations using DPIs. DPIs also have several advantages in drug delivery to the lungs, which include high-dose delivery, propellant-free delivery, portability, ease of operation as well as low-cost devices. Besides, DPI formulations generally display enhanced stability of the active incorporated ingredients as they are present in the form of dry powders [32]. DPIs are classified into different categories depending on the number of deliverable doses of the device, the patient contribution to aerosolizing the powder and the mechanism of powder dispersion. Based on these criteria, DPIs can be classified into three categories, namely the breath-actuated single-dose devices, the breath-actuated multi-dose and active devices. One of the characteristics of formulating DPI deliveries for pulmonary drug delivery includes the drug particles need to have mass median aerodynamic diameter (MMAD) smaller than 5 µm, whereas a MMAD of 1–3 µm is necessary for targeting deposition in smaller airways. The *Aspergillus* conidia are able to penetrate deep into the lungs and hence, the antifungal drug particles also needed to penetrate as deeply as the inhaled conidia. Further, the powder blends in DPI can either be a mixture of micronized drug with coarse carrier particles such as lactose, mannitol and sucrose or drug particles encapsulated in nanocarrier particles [31,33].

##### Micronized Drugs for Lung Delivery against Fungal Infection

DPI formulations of antifungal agents were found to be effective in curbing the systemic toxicities as targeted local delivery of drug can be achieved and thus, a high systemic concentration is not needed to achieve an adequate pulmonary concentration of the drug. In one study, DPI formulations of AmB were formulated with cholesteryl carbonate esters (CCEs), which include sodium cholesteryl carbonate (SCC), cholesteryl palmityl carbonate and dicholesteryl carbonate by a solvent evaporation technique. The developed AmB-loaded DPI formulations with CCEs (AmB-CCE) had higher reported in vitro drug release when compared to AmB-cholesterol because CCEs act as efficient solubilizer for AmB in the aqueous phase of the formulations. AmB, being a hydrophobic drug molecules prefers to stay in the liquid crystal phase rather than the aqueous phase but the drug release was dependent on the extent of AmB dissolution in the aqueous phase. Due to the presence of CCEs, the environment in liquid crystal phase became less flexible and more packed, which leads to reduced dissolution of AmB in the liquid crystal phase, which in turns increases the solubility of AmB in the aqueous phase. The developed AmB-CCE with the best aerodynamic properties formulated with SCC showed MMAD of 3.8 ± 0.7 µm, emitted dose (ED) of 87.9 ± 1.3%, fine particle fraction (FPF) of 38.0 ± 1.3% and geometric standard deviation (GSD) of 3.02 ± 0.62. All the DPI formulations of AmB-CCE demonstrated greater in vitro antifungal activity against *Crytococcus neoformans* (ATCC 90113) and *Candida albicans* (ATCC 90028) as evidenced through the 2 to 4 times lower minimum inhibitory concentration (MIC) and minimum fungicidal concentration (MFC) compared to pure AmB. This was due to the synergistic effect provided by the liquid crystals with AmB in the inhibition of fungal growth as well as the facilitation of transfer or induction of ionophore formation as AmB drug molecules may be transported into the fungal cells together with the liquid crystal molecules [34]. The improvement of antifungal activity and aerosol performance of the DPI formulations using CCEs demonstrated their suitability to be used as carriers.

On the other hand, studies were also conducted to investigate the development of DPI formulations for ITZ. In a study by Duret et al., solid dispersions (SD) of ITZ were prepared by a spray-drying method with mannitol as carrier and tocopherol polyethylene glycol 1000 succinate (TPGS 1000) as surfactant. The produced ITZ-SD showed better aerosol performance when compared to bulk ITZ with FPF of 47 ± 2%, ED of 53 ± 2% and MMAD of 1.61 ± 0.05 µm. This is due to the ability of mannitol to prevent particle agglomeration and aggregation by capillary interactions during inhalation, which could decrease the flowability of the dry powder. Besides, mannitol also has the ability to stabilize the amorphous form of ITZ in the formulation, which may contribute to the better aerosol performance of the formulation. Dissolution and solubility profile of a pulmonary drug delivery formulation is an important characteristic as the pharmacological activity of the antifungal agent depends on the level of the dissolved drug in the thin layer of the lung surfaces’ surfactant fluid. The use of TPGS 1000 and mannitol improved the in vitro dissolution or solubility profile of the ITZ-SD as evident from the 4 to 6-fold greater saturation level of ITZ in the physiological phosphate buffer when compared to the unformulated ITZ. This improvement of ITZ saturation solubility in the formulation could be attributed to the ability of TPGS 1000 in reducing re-crystallization of ITZ amorphous particles in the dissolution medium by intermolecular interactions and the ability of mannitol to quickly dissolve in contact with the dissolution medium, enhancing the surface area, which ultimately accelerated dissolution of the amorphous ITZ particles. Both TPGS 1000 and mannitol improved the wettability of the drug particles and saturation solubility of the poorly water-soluble ITZ. However, addition of TPGS 1000 into the formulation negatively impacted the aerosol performance of the DPI formulations which was evident from the increment of MMAD from 1.61 ± 0.05 µm to 4.5 ± 0.6 µm and reduction of FPF from 47 ± 2% to 16 ± 2% [35]. Nonetheless, both studies demonstrated the possibility of reduced dosage of antifungal agents by the micronized DPI formulations, which can subsequently reduce the total systemic exposure of antifungal agents. Furthermore, the use of TPGS 1000 improved the dissolution and saturation solubility of ITZ-SD, however, too much TPGS 1000 can decrease the aerosol performance of the SD due to its surface accumulation and softening effect during the spray-drying process [35]. Hence, the same research group later developed SD of ITZ with mannitol and hydrogenated soy-lecithin (ITZ-SD2), a surfactant present in the lung. The use of hydrogenated soy-lecithin showed a different result in aerosol performance of the ITZ-SD2 when compared to TPGS 1000 as the flow properties of the formulations were improved in presence of the surfactant. This might be due to the ability of hydrogenated soy-lecithin in reducing the SD particles surface energy, water sorption, cohesion and hence, improve the device emptying of the drug particles. Besides, the surface irregularities and particle size growth induced by the hydrogenated soy-lecithin could make the inter-particular distance longer and reduce the total surface area, which results in the reduction of interaction between the particles. The developed ITZ-SD2 with hydrogenated soy-lecithin showed improved dissolution profile when compared to raw crystalline ITZ due to improvement of wettability of ITZ by mannitol and hydrogenated soy-lecithin. Interestingly, there was a linear relationship between the surfactant content and the ITZ dissolution rate where increasing surfactant content proportionally accelerates the ITZ dissolution rate. Modulation of release properties of the formulations could be achieved by adjusting the surfactant content to provide desired in situ pharmacokinetic profiles [36].

Additionally, introduction of phospholipid in DPI formulations has been shown to enhance the dissolution profile of poorly-water soluble antifungal agents. However, the improvement of dissolution rate of the antifungal agent may not be a favourable trait for drug delivery through the pulmonary route, as extended lung retention is needed for adequate fungicidal activity near the infected lung area. A study by the same group, Duret and team, reported the development of ITZ DPI formulations using spray-dried mannitol with or without phospholipid [37]. All the ITZ DPI formulations developed had MMAD of around 2 µm and sizes between 3.9 and 5.59 µm. This allowed good penetration of the particles into the lungs of the mice after insufflation. It was found that the incorporation of phospholipid in the formulation enhanced the ITZ dissolution rate significantly as compared to the ITZ formulation without phospholipid due to improved wettability [37]. The improvement of drug solubility and dissolution might be due to the formation of amorphous ITZ, which is widely dispersed within the mannitol particles in crystalline state in addition to the wetting enhancement provided by phospholipid [36]. Through pharmacokinetic studies in murine models using male outbred ICR mice, it was demonstrated that the presence of phospholipid further improved ITZ wettability and accelerated the absorption of ITZ by the lung epithelium. This was due to the ability of the phospholipid to induce cell membrane modifications, which include decreasing bilayer stability, affecting tight junctions and thus, accelerating paracellular absorption of the drug particles. Due to this accelerated drug absorption, ITZ was eliminated from the lung faster and the total lung exposure was reflected through the lower half-life of ITZ (4.1 h) for ITZ-SD with phospholipid when compared to ITZ-SD without phospholipid (14.7 h). Alternatively, the ITZ-SD without phospholipid had slower absorption of drug with extended lung retention and higher bioavailability in the mice with AUC_0–24h_ in lungs of 332.6 µg.h/g than ITZ-SD with phospholipid 143.8 µg.h/g [37]. Hence, it was important to create a balance between drug dissolution rate and bioavailability while utilizing a wettability enhancer such as phospholipid in the DPI formulations to obtain desired lung retention profile in the DPI formulations.

Other than spray-drying method, the thin film freezing (TFF) is another method used in producing DPI formulations. A study by Beinborn et al., developed VRZ DPI formulations using this TFF method. They demonstrated that microstructured crystalline VRZ DPI formulation produced without the use of polyvinyl pyrrolidone K25 (PVP K25), a polymeric stabilizer, displayed better aerodynamic properties than nanostructured amorphous formulations. Although the nanostructured amorphous TFF-VRZ-PVP 25 displayed a greater dissolution rate than the microstructured crystalline TFF-VRZ formulation with 1.3 times higher area under the dissolution curve, TFF-VRZ has higher FPF (37.8% to 32.4%) and smaller MMAD (4.2 µm to 5.2 µm) than the TFF-VRZ-PVP K25. The less favourable aerosol properties of TFF-VRZ-PVP K25 can be due to PVP K25 allowing the formation of nanostructured amorphous formulations of VRZ which result in less respirable particles due to difficulty in breakage of the low-density aggregate particles as they exit the punctured capsule during aerosolization. This can be attributed to the strength and elasticity of the bridges between the drug particles added by the polymeric stabilizer. Besides, the addition of polymeric stabilizer might cause adherence of particles to the induction port of the impactor apparatus due to enhanced hygroscopicity and electrostatics of the amorphous formulation and thus, negatively affect aerosolization of the antifungal drug particles. For the pharmacokinetic parameters tested in male outbred ICR mice, TFF-VRZ had achieved higher lung bioavailability of VRZ with AUC_0–24h_ of 452.6 µg.h/g as compared to TFF-VRZ-PVP K25 with AUC_0–24h_ of 232.1 µg.h/g. Besides, the microstructured crystalline TFF-VRZ also demonstrated longer retention than nanostructured amorphous TFF-VRZ-PVP K25. This resulted in accelerated dissolution of nanostructured amorphous TFF-VRZ-PVP K25 than microstructured crystalline TFF-VRZ in the lung lining allowing less time for interaction with the fungal pathogens. Moreover, the antifungal agent appeared to be eliminated from the plasma more slowly for the microstructured crystalline TFF-VRZ, probably due to the large reservoir of VRZ in the lung tissue as compared to TFF-VRZ-PVP K25, where the VRZ has dissolved much quicker and absorbed into the systemic circulation. Hence, the microstructured crystalline TFF-VRZ formulation was reported to be more favourable for pulmonary delivery of the antifungal agent as it can achieve high and prolonged concentrations in the lungs with low systemic bioavailability [38]. Although the use of polymeric stabilizer such as PVP can improve the dissolution of poorly soluble antifungal drug, the enhanced solubility of drug in pulmonary area may not be favorable in terms of lung retention.

In order to further prolong the pulmonary residence time of drug in the lungs, formulations with sustained release properties should be considered. In 2015 Arora et al. prepared sustained release inhaled dry powder formulations of poly(lactic acid) (PLA) microparticles containing VRZ (VLM) and determine their appropriateness to be used in targeting lower part of lungs. Based on the results, high entrapment efficiency (EE) of the VLM was obtained, which was around 99% with a drug loading (DL) of approximately 20%. The VLM exhibited as small spherical, amorphous particles with size around 2.4 µm, which was smaller than the spray dried VRZ. Both VLM and VRZ showed low moisture absorption. VLM had MMAD of around 3.68 µm, which was smaller than VRZ, GSD of 2.13 and had higher emitted FPF of around 43.56% than the VRZ. Normally, monodisperse aerosols have a GSD of 1 whereas, a GSD higher than 1.22 is considered as polydisperse aerosol. Most therapeutic aerosols have GSD values between 2–3 and are considered polydisperse. FPF, meanwhile, is the measurement of the percentage of emitted particles with size below 5 µm [39]. The larger MMAD and lower emitted FPF of VRZ might be caused by the crystalline structure of VRZ, which increases the cohesion of particles. The authors concluded that the exhibited aerosolization performance of VLM could be suitable for inhalation administration with sustained release properties. The sustained release properties of the formulation were due to the slow release of drug from the controlled release polymer, PLA, where the drug was entrapped within the polymer matrix [40].

In another study by the same research team, Arora et al. developed inhalable large porous microparticulate dry powder formulations containing VRZ (VLPP) using PLA, poly-lactide-co-glycolide (PLGA) 752H or PLGA 502. However, focus was shifted towards VLPP fabricated from PLA as it had well-defined morphology, whereas, the VLPP fabricated from PLGA 752H had a rough surface whereby broken structures were observed in the VLPP fabricated from PLGA 502 which might be due to the lesser intrinsic strength of PLGA 502 and an inability to maintain the integrity of the high porous particles. The developed and optimized VLPP formulation exhibited MMAD of around 2.85 µm and FPF of around 27.3%, which were perfect for inhalation. The authors further explained that the FPF could be correlated to the higher surface energy of the VLPP that had increased the cohesion between the particles and formed larger loose aggregate, which could experience higher shear force leading to efficient de-agglomeration. In addition, homogenization speed during the preparation process, porogen concentration and polymer types also showed an effect on the aerosolization performance of the VLPP. VLPP had EE of around 84% and showed sustained release properties with a maximum 95.34% of drug release over 7 days. Extended action of VLPP at the action site can be expected as its larger size (geometric size of 8–13.5 µm) makes it able to evade the uptake of macrophages. It was found that the geometric size of the particles was greatly dependent on the homogenization speed and porogen concentration. Overall, the study showed that the formulated VLPP could be suitable for the treatment of IPA. Utilization of PLA and PLGA in both studies produced porous particles which improved the aerodynamic performance of the DPIs [41]. As the FPF of VLPP was just above the desired range, the same group developed another inhaled VRZ microparticle dry powder formulation with the addition of leucine as dispersibility enhancer using a spray drying technique. Introduction of leucine in the formulation was reported to further improve the aerosolization performance of the dry powder formulation. According to the results obtained, the authors observed that as the concentration of added leucine was increased, the size distribution of the particles decreased. This might be due to the ability of leucine, which is able to accumulate at the air-liquid interface owing to their hydrophobic structure. Besides, the developed formulation exhibited high EE (99%). Moreover, the developed dry powder with MMAD of around 3.79 µm and FPF of around 60% were found suitable for delivery through inhalation. The authors further concluded that the aerosolization performance of the developed microparticle formulation was improved significantly, which may be due to the present of leucine. A 3-month stability assay showed that the developed formulation was able to remain stable under room conditions. In addition, in vitro drug deposition and diffusion assay performed on calu-3 cells model showed that the 98% of developed dry powder formulation was transported across lung epithelial cells in 4 h indicating the transport mechanism of VRZ was not affected by the addition of leucine. Furthermore, higher lung exposure of VRZ was noted through pulmonary delivery of the developed formulation compared to intravenous administration of free VRZ, indicating higher efficacy of the drugs, whereas, around 2-fold higher VRZ concentrations were found in the spleen, liver and kidney after the treatment with intravenous VRZ, indicating the leucine-modified VRZ was deposited less in other organs. Hence, inhalation administration of leucine-modified VRZ can increase the concentration of VRZ specifically in the lungs [42].

Other than that, a study carried out by Karashima et al. used a jet-milling system to formulate cocrystal micronized dry powder formulations of ITZ. Cocrystals of drugs are combinations of drug together with aliphatic dicarboxylic acids such as tartaric acid (TA), succinic acid (SA), fumaric acid (FA) and malic acid (MA). The solubility and dissolution rate can be improved by the formation of cocrystal. The mean diameter of the micronized ITZ cocrystals with SA (ITZ-SA) and micronized ITZ cocrystals with TA (ITZ-TA) including spray-dried mannitol as the dry powder carrier were 2.9 µm and 3.9 µm, suggesting high efficiency in inhalation delivery. The ITZ-SA and ITZ-TA had a DL of approximately 19.3 and 18.3%, respectively. The ITZ-SA showed a higher dissolution rate when compared to pure ITZ formulation, amorphous ITZ and other ITZ cocrystals. The higher dissolution rate of ITZ-SA might be due to the higher disorganization in the triazolone ring of the ITZ-SA, which can be reflected through its lower melting point. Apart from that, improved dissolution rate increased the area under the curve (AUC) and bioavailability of ITZ in the Sprague-Dawley rat pharmacokinetics assay when compared to the crystalline ITZ and amorphous ITZ [43].

On the other hand, drug nanoparticle formulations of micronized drugs have been developed to further improve the DPI formulations of the micronized drug for efficient lung deposition and rapid dissolution of poorly water-soluble drugs. ITZ nanocluster DPI formulations were developed by a wet-milling technique with ethanol. The selection of ethanol as solvent improved the solubility of ITZ as ethanol enhances the wettability and dispersibility of the ITZ during milling. Besides, ethanol has the ability to shift the physical characteristic of ITZ by facilitating recrystallization, controlling the crystallinity of ITZ during the milling process, which provide greater physical stability. The ITZ nanocluster DPI formulations demonstrated better aerosol performance than micronized ITZ due to smaller particle size and higher porosity of the milled ITZ of nanocluster formulations. However, the aerosol performance of the optimized ITZ nanocluster formulations showed some dependence on flow rate, where the milled ITZ showed best aerosol performance at a flow rate of 90 L/min. The optimized ITZ nanocluster DPI formulation was found to possess MMAD of 1.2 ± 0.1 µm, FPF of 91.8 ± 1.2%, ED of 80.3 ± 10.7% and GSD of 2.0 ± 0.1 [44].

The results obtained from this study were in contrast with those of a study conducted by Beinborn et al., which may be due to the selection of excipients in developing the nanostructured DPI formulations [38]. Depending on the interactions and properties of the excipients used, the nanostructured formulations produced may have improved or deteriorated aerosol performances as compared to microstructured formulations. Through the formulations mentioned above, dissolution of the poorly water-soluble antifungal drugs can be enhanced using solubilizer and stabilizers such as carbonyl esters, dicarboxylic acid and PVP whereas the aerodynamic properties of the DPI formulations can be improved with sugar carriers such as mannitol and anti-adherents such as leucine. The micronized dry powder inhaler formulation approaches intended to be used in the treatment of PA are summarized in Table 1. Beside the approaches mentioned, pulmonary drug delivery using drug nanoparticle formulations can also be implemented as nanocarriers for lung delivery.

##### Nanocarriers for Lung Delivery against Fungal Infections

Nanocarriers can serve as the suitable drug carrier systems for pulmonary fungal infections due to their high surface area to volume ratio and the ability to enhance the bioactivity of the antifungal drugs. As they generally have smaller size (<500 nm), they can penetrate into alveoli cavities and thus, allow optimal antifungal deposition at the target site [29,45]. Besides, they have enhanced permeability and retention which can be beneficial for site-specific drug delivery and controlled drug release through improved solubility, prolonged duration of action and providing protection from degrading enzymes [46]. This allows lesser dose of antifungal agents to be administered and thus, reducing systemic exposure of the drug which can reduce the incidence of drug toxicity associated with azoles (especially hepatotoxicity) [47,48]. Nanocarriers can either be lipid-based or polymer-based but lipid-based nanocarriers are preferred due to their biocompatibility and biodegradability. Lipid-based nanocarriers consist of liposomes, lipid micelles, nanoemulsions, solid lipid nanoparticles (SLN) and nanostructured lipid carriers (NLC) [47]. Few of the examples of nanocarrier based lung delivery using dry powder inhaler platform intended to be used in the treatment of PA are summarized in Table 2 and discussed in detail in hereafter.

Lipid-based delivery for delivering drugs in lungs:

Liposomal delivery approach to delivering drugs in lungs

Liposomes are bilayered vesicles with an aqueous core surrounded by phospholipid membranes. Their usage in drug delivery can be dated back to the beginning of 1980s and they exhibit the advantage of encapsulating various drug entities, both hydrophilic and hydrophobic, as well as intrinsic biocompatibility [30]. Liposomes confer a variety of advantages as drug carriers such as enhancing the bioavailability and reducing systemic toxicity of the encapsulated drug. A liposomal formulation has been developed for AmB (AmBisome^®^) and it is the first successful commercial nanoformulation of an antifungal drug. The liposomal AmB has reduced nephrotoxicity when compared to AmB deoxycholate injection due to the prolonged circulation of small size liposomes (<100 nm) which allows distribution into other organs. Besides, the AmB in liposomes are selectively transfer into fungal cells with minimized uptake by the human cells [49]. However, the conventional liposomal formulations have the tendency of drug leakage from the liposomes during circulation, extensive uptake of the liposomes by the RES as well as inability of extravasation into the infected lung tissues. Hence, liposomal DPI (LDPI) formulations were ventured into in seek of stabilizing the liposomal system and localized liposomal drug delivery [50].

A study by Shah et al. reported LDPI formulations of AmB with hydrogenated soy phosphatidylcholine (HSPC), cholesterol (CHOL) and either saturated soy phosphatidyl glycerol (SPG-3) or stearylamine prepared by a modified reverse phase evaporation technique. Formation of stable emulsions by this combination produced multilamellar liposomes, which had a high percentage of drug entrapment (80%). The formation of good vesicles also lead to less drug leakage as compared to conventional liposomal formulations, which could be contributed by the cryoprotective effect of the crystallized sugar. The LDPI formulation of AmB also demonstrated efficient detachment of the liposomal drug from the carrier as the high-energy adhesion sites are occupied by the sugar while the liposomal drug particles are attached to the low-energy adhesion sites left and thus, result in higher FPF (22.5 ± 2.2%). Due to the efficient detachment, the developed LDPI AmB possessed good flowability and floodability according to the performance in angle of repose (28.3 ± 0.6°), dispersibility index (20.8 ± 1.0), compressibility index (23.5 ± 1.8) and effective index (44.8 ± 1.6) as compared with control, Ashthalin^®^ with FPF of 27.1 ± 2.0% and effective index of 48.6 ± 1.7. The LDPI formulations also showed great stability at refrigerated and controlled room temperature storage conditions due to the reduction of rate of hydrolysis or oxidation of drug in anhydrous state by the association of CHOL with PC and drug at molecular level. Besides, re-encapsulation of liposomes occurs due to higher proportion of amphiphilic PC in the formulations also contributed to enhanced stability at the accelerated storage condition [50]. Later, a study by the same group prepared LDPI formulations of AmB by sieving the porous cake of liposomes obtained after lyophilization. The fines produced were added in the LDPI formulations in different percentages and the sequences of addition of fines were investigated. In this study, the LDPI formulation of AmB was optimized in terms of concentration of carrier as an optimum concentration of carrier is required to achieve detachment of liposomal drug from the carrier molecules [51]. The introduction of fines improved the aerodynamic properties of the LDPI formulation as fines increase the tensile strength of the bulk powder and thus, the aerodynamic drag forces required to fluidize the cohesive powder also increased. This leads to better dispersion performance of the LDPI formulation together with the effect of particle-particle and particle-wall collisions. However, too high amounts of fines can make the particles get entrained as stable agglomerates, reducing the aerodynamic properties of the LDPI formulation [52]. Hence, an optimum percentage of fines was determined in the study. The optimized LDPI formulation of AmB has FPF of 28.4%, dispersibility index of 31.8 and effective index of 50.4. Interestingly, LDPI formulation with negatively-charged liposomes demonstrated more effective liposomal drug deposition in to lung suggesting the charge generation in liposomal powder during dispersion, which can affect the lung deposition efficacy [51]. These studies demonstrated more stable liposomal formulations of antifungal agents in DPI as compared to conventional liposomal antifungal formulations.

Other formulation approaches to deliver drugs in lungs

Transfersomes are modified deformable liposomes where edge activators are included in the liposome composition. The addition of edge activators increased the deformability of the liposomes due to their ability to weaken the lipid bilayers of the vesicles. This leads to enhanced drug penetration [30]. A study by Aghdam et al., developed an ITZ-loaded nanotransfersomal DPI formulation where the nanotransfersomal formulation was optimized with lecithin:Span^®^ 60 in the ratio of 90:10 and co-spray dried with mannitol. It had been reported that the particle size of the nanotransfersome vesicles were not significantly affected by the type of surfactant (lecithin, mannitol, ursodiol, Span^®^ 60 and Span^®^ 80). The ITZ-loaded nanotransfersomemal DPI formulation has suitable aerosolization performance evident from the values of MMAD (5.1 ± 0.7 µm), FPF (37.3 ± 3.1%), emission (82.7 ± 2.6%), dispersibility (45.23 ± 2.9) and GSD (2.1 ± 0.2). This may be due to the utilization of spray drying technique for stabilization of nanoliposomes, which resulted in the development of uniform size particles with good aerosolization performance. It was predicted that through pulmonary drug delivery, once deposited in the aqueous lining fluid in the lung, the nanotransfersomes aggregate. The mannitol in the formulation will quickly dissolve providing a large surface area for the dispersion of nanotransfersomes [53].

In the literatures reviewed earlier about the lipid-based nanocarrier DPI formulations, the improvement of the aerosol performance can be attributed to the use of sugar carrier (mannitol) and fine particles, which improved the detachment of the drug particles from the high energy adhesion sites. This is in consistent with the improvement of aerodynamic performance of the formulation in the study by Duret et al. using mannitol as sugar carrier [35].

Polymeric drug delivery systems to deliver drugs in lungs

In today’s modern drug delivery system era, polymers play an important role in the pharmaceutical field and are being utilized widely in improving the pharmacokinetics of drugs. Polymeric drug delivery system mainly provide sustain release properties by aiding the release of hydrophobic and hydrophilic drugs in a controlled manner over an extended period at the targeted sites [54,55].

There are several models to describe the release kinetics mechanisms of drugs from polymer-based carriers. Diffusion-controlled drug release happens when the drug is dispersed or dissolved in a core surrounded by a rate-limiting membrane. Another type of diffusion-controlled release occurs when the drug is dispersed evenly in the rate-limiting polymer matrix, whereby the drug will be released at a high rate initially and this rate will decrease over time due to the increase of diffusion distance. Solvent-controlled release describes the swelling of the system when absorbing water which increases the mesh size of the polymer and releases the drug. Finally degradation-controlled release describes the release of drug when a biodegradable polymer degrades [56]. By maintaining the drugs concentration within the therapeutic range, polymeric drug delivery systems improve the efficacy and safety of drugs as well as reduce the dosing frequency, increasing patient compliance [54,57,58]. Apart from that, properties including biodegradability, high loading capacity, biocompatibility, non-toxicity and prevention on the degradation of drug are gaining the attraction of many researchers for the development of polymeric drug delivery systems [54].

Polymeric nanoparticles are novel nanocarriers that protect therapeutic agents from hydrolytic or enzymatic degradation and deliver the drugs to the targeted site in the treatment of fungal infections. Higher amounts of drug can be accumulated at the targeted site when polymeric nanoparticles are used as carrier as their small sizes allow easier penetration into the cells [59]. Based on the structure and composition, polymeric nanoparticles can be divided into two forms, which are nanospheres and nanocapsules. Nanospheres are characterised as a spherical mass of solid polymer matrix in which the therapeutic agents are dispersed homogeneously within the sphere or adsorbed on the surface. On the other hand, nanocapsules have a vesicular structure and act as a reservoir system. The therapeutic agents are embedded in the interior cavity of nanocapsules and enclosed by the polymeric membranes. Nanocapsules have several advantages over nanospheres. Nanocapsules allow therapeutic agents in both aqueous and solid forms to be incorporated in the cavity. Moreover, lesser quantity of polymers is used to produce nanocapsules. Thus, potential toxicity will be reduced [60]. Lately, aspects of different antifungals incorporated with polymeric nanoparticles are being investigated extensively by researchers.

Jafarinejad et al. prepared a formulation of ITZ polymeric nanoparticles with chitosan as cationic polymer (Ch-ITZ) and tripolyphosphate (TPP) as anionic entity using an ionic gelation method. Ch-ITZ was further formulated with leucine, mannitol and lactose into dry powder formulations for pulmonary delivery using a spray drying method. The prepared formulation had high drug EE of 55% in 1:3 of chitosan: TPP with particle size around 240 nm and zeta potential of around 15.33 mV. The two times larger amount of TPP developed strong electrostatic interaction between the two opposite entities which leads to the formation of nanoparticles and improves the EE. The prepared formulation showed an instantaneous release of 80% of drug during the initial 4 h followed by controlled released of the remaining drug over 48 h. Ch-ITZ with the addition of 10% mannitol and 10% leucine showed high FPF of around 42.9% and emitted dose of around 71.8%, which rose the deposition of ITZ in the lungs [61], whereas, Ch-ITZ with the addition of 10% of mannitol only exhibited FPF and emitted dose of around 33% and 59.4%, respectively, indicating that the aerosol performance of the Ch-ITZ improved with the addition of leucine as it increased the roughness of the particles, decreased the contact area of particles and decreased the cohesion between particles [62]. Together with the action of mannitol stated in the previous study, incorporation of leucine and mannitol provide synergistic action to improve the aerosol performance of Ch-ITZ for pulmonary delivery [35].

**Figure 3:**
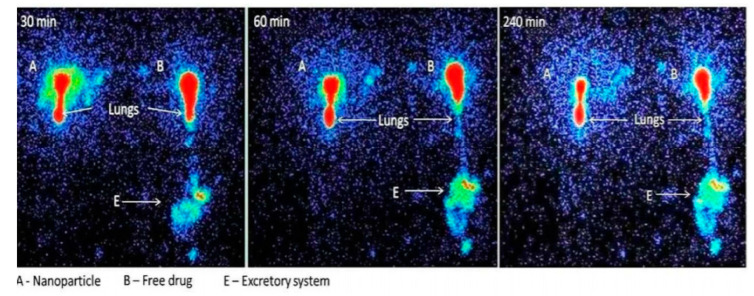
Gamma scintigraphy showing that 4 h post administration of free voriconazole accumulated in the excretory system whereas VNP remained in the lungs [64].

Alternatively, Sinha et al. developed a dry powder formulation of VRZ-loaded PLGA nanoparticles (VNP) for pulmonary delivery. The researchers formulated the VNP in porous and non-porous forms using a multiple-emulsification method. Maximum DL and EE obtained by the VNP were around 30% and 62.8%, respectively. It was found that EE per unit weight of drug is inversely proportional to the amount of drug added as a higher concentration of drug in the polymeric matrix will increase the diffusion of the drug out from the polymeric matrix. Most of the formulated batches exhibited particles with average diameters under 400 nm. The zeta potential of VNP of around −20 mV indicates the formulations need to be stored in lyophilized state and reconstituted before use. The developed VNP showed a sustained release property in the study with instantaneous release of 20% of the drug, followed by sustained release over 15 days. In comparison to non-porous VNP, porous VNP had a higher drug release rate than the non-porous VNP. This is due to the larger surface area of porous VNP, which enables the diffusion of drugs. However, the result was not significant as there was a capillary force of the liquid that flowed against the direction of drug diffusion, which needed to be surmounted. Porous VNP also had lower MMAD thus showing deeper penetration into the lungs than the non-porous VNP. Additionally, the maximum drug deposition of porous VNP and non-porous VNP were 120.38 µg/g and 74.83 µg/g and could be detected after 7 days and 5 days post-administration, respectively. Uniform distribution of both the porous and non-porous VNP across the lung tissues was indicated by monitoring fluorescence-labelled VNP. Overall, this study suggested the sustained release VNP is another option in the delivery of antifungals [63]. A further study on VNP was carried out by Das et al. to investigate the release profile of VNP in the lungs in vivo by radiolabelling the particles with technetium-99m and assessing their distribution using gamma imaging. The particles of VNP had a smooth surface with average size of 300 nm and negative zeta potential between 8–11 mV. This negative zeta potential was mainly due to the hydrogen ions separated from the carboxyl functional groups of PLGA. Two similar formulations with different DL of 2.91% and 2.57% respectively were mainly due to the higher homogenization speed that produced larger shear force, which results to faster evaporation of solvent and diffusion of water and reduced the partition of drug out to the aqueous medium. The in vitro release kinetics of the VNP in simulated lung fluid followed both Higuchi kinetics and the Korsmeyer-Peppas model indicating the VNP was released via a combination of diffusion and erosion mechanisms, which is also known as non-Fickian diffusion or anomalous diffusion. After pulmonary administration of VNP dry powder, VNP showed a higher rate of retention in the lungs as compared to free VRZ. This might be due to the ability of VNP to deposit in the deeper regions of the lungs where epithelial cells with tight junctions and alveolar lining with various lipids as well as proteins act as the barrier preventing the transportation of nanoparticles, whereas, free VRZ that is deposited in the lungs may move to the lung epithelial cells and further translocate to the blood resulting in pulmonary clearance (Figure 3). Hence, the study showed that pulmonary delivery of VNP is more efficient than that of the free drug [64].

PLGA-based nanoparticles such as VNP have been widely explored as carriers to deliver drugs to the targeted site. However, modifications of the surface of PLGA-based nanoparticles may bring additional advantages such as improvement in cellular adhesion, increased residence time at the targeted site and inversion of zeta potential. In this context, Paul et al. formulated chitosan-coated PLGA nanoparticles containing VRZ (Ch-VNP). The particles of Ch-VNP had spherical smooth surfaces with sizes around 300 nm. The authors also found that there was no significant chemical interaction between the excipients and the drug. Ch-VNP had comparable EE to the VNP of around 70% but its DL was lower than the VNP, which was due to the coating process that results to the loss of drug. Furthermore, higher colloidal stability was expected in the Ch-VNP compared to the VNP due to the positively charge chitosan inverse the zeta potential of Ch-VNP to +31.9 mV while VNP had zeta potential of −14.8 mV. A zeta potential closer to 0 mV indicates less electrostatic repulsion between the particles, thus resulting in precipitation. An in vitro drug release assay showed that the Ch-VNP instantaneously released the drug during the first 24 h followed by a sustained release manner over 8 days. The MMAD, GSD and FPF of the Ch-VNP were around 2.8 µm, 3.04 and 57.47% respectively. In vivo assays performed on Swiss albino mice showed that the maximum concentration achieved by the non-coated VNP was higher than with Ch-VNP, which was due to the slow diffusion of the hydrophobic VRZ from the hydrophilic chitosan that resulted in slower drug release of Ch-VNP. Nevertheless, higher AUC in the lungs after pulmonary administration of dry powder Ch-VNP revealed that higher bioavailability was achieved compared to the free VRZ and VNP. This might be due to the fact chitosan increased the bio-adhesion of the particles. In turn, the bio-adhesion increases the residence time of Ch-VNP (mean residence time of around 300 h) in the lung epithelial cells and enhances the drug absorption. This was proved by gamma scintigraphy imaging which showed higher signals in the lungs compared to the VNP. The slow release of drug is also responsible for the enhancement of bioavailability of Ch-VNP. Therefore, the study showed that the enhanced bioavailability of Ch-VNP in the lungs is useful for the treatment of pulmonary fungal infections [65]. From these studies, it generally demonstrated the sustained release property is a feature for polymeric-based nanocarrier formulations due to the slow release of antifungal drugs from the polymer matrix.

#### 3.1.2. Nebulisation Approach to Deliver Drugs in Lungs

Nebulizers work by converting liquid formulation of drugs into a vapour for inhalation. Nebulizers offer various advantages in delivering drugs to patients such as coordination of patients between actuation and inhalation is not necessary. Secondly, in comparison to other inhalation devices, nebulizer is able to deliver larger amounts of drug to the patients. However, this delivery system is associated with several disadvantages, which includes time-consuming operation, bulky appearance, nonportable nature, contamination issues of the delivery content, wastage of drug in the environment, relatively expensive, wide performance variation between different models and operating conditions with poor delivery efficiency [66]. There are three types of nebulizer, including mesh nebulizers, ultrasonic nebulizers as well as jet nebulizers, where mesh nebulizers are the latest innovation. These mesh nebulizers allow medication in liquid form to pass through the mesh to generate consistent aerosol droplets that can inhaled comfortably. Ultrasonic nebulizers utilise ultrasonic frequency to generate vibrations on a metal plate which convert a liquid medication into a mist for inhalation, whereas, jet nebulizers generate aerosol droplets based on the Bernoulli principle [31]. However, nebulizers have the limitations of difficulty in cleaning, longer duration of treatment and drug loss during expiration [67].

##### Nebulization of Intravenous Formulation

In order to develop nebulizer formulations of antifungal agents, commercially available VRZ IV formulation containing 100 mg/mL sulfobutyl ether-β-cyclodextrin sodium was aerosolized for nebulization in 2009. According to the results of in vivo study in *Aspergillus fumigatus*-infected outbred ICR mice treatment with aerosolized VRZ has significant survival advantages (92%) as compared to treatment with AmB (25%). This improved survival rate in the infected mice might be attributed to the reductions in the extent of invasive PA with the administration of aerosolized VRZ. Besides, the aerosolized formulation was also well tolerated with the absence of lung injury or inflammatory changes based on histological findings in uninfected mice [68].

This successful targeted pulmonary drug delivery encouraged the adaptation of the commercially available IV formulation with the requirement of further evaluation on the efficacy of aerosolized formulations. Therefore, later in 2019, VRZ lyophilized powder was reconstituted with sterile water for injection while PCZ IV solution was diluted with 0.9% normal saline. Both reconstituted or diluted formulations were then aerosolized for nebulization. Both aerosolized azoles possessed favourable aerodynamic properties with MMAD of 2.8 µm, FPF of 76.8% and GSD of 2.0 for aerosolized VRZ, where MMAD of 2.4 µm, FPF of 62.9% and GSD of 2.4 for aerosolized PCZ with lower concentration (6 mg/mL). Besides, both aerosolized azoles had high respirable fraction, which was less than 5.4 µm (>75%) indicating possible deposition in the peripheral airways of the lungs [69]. On the other hand, aerosolization of reconstituted 100 mg powder of Mycamine^®^ (micafungin) and Ecalta^®^ (anidulafungin) with 0.9% sodium chloride possessed suitable pH, osmolality, chloride content and density values, similar to the values of water. Besides, the aerosolized formulations do not contain any excipient, which are associated with the production of cough or bronchoconstriction, suggesting less withdrawal of treatment by patients. However, further evaluation on aerodynamic properties and clinical characteristics should be carried out to ensure the effective dissemination of the antifungal agents in the lung [70]. These studies demonstrated that the suitability of commercially available IV formulations of antifungal agents can be aerosolized for nebulization and encouraged further studies on different commercially available antifungal formulations. In addition, further in vivo pharmacokinetics studies are required to be carried out to ensure the efficiency and safety of nebulized IV formulations of antifungal agents. The aerosolized intravenous formulation approaches for nebulization for the treatment of PA are summarized in Table 3.

##### Nanocarriers for lung delivery against fungal infection for nebulized delivery:

Lipid-based delivery systems for nebulization:

Liposomal approach to deliver drugs in lungs using nebulization techniques

It could be understood from the discussion in the previous section that aerosolization of antifungal agents is one of the strategies to treat fungal infections of lung origin. In this context, the aerosolization of liposomal formulation showed the possibility of targeted drug delivery through the pulmonary route. A study reported on preparation of a liposomal formulation of AmB with egg phosphatidylcholine (PC) and CHOL modified with coating of O-palmitoyl mannan and O-palmitoyl pullulan. Mannan and pullulan were selected as targeting ligands in the formulation as they have specific affinity for macrophage mannose receptors, which are known to allow targeted delivery of AmB to the lungs and specifically to pulmonary alveolar macrophages. The developed liposomal AmB formulation had a mean vesicle size of 2.56 ± 0.32 µm. In the airways’ penetration efficiency test, the aerosolized liposomal AmB showed a lower quantity of drug retained on the filter, which could be due to the retention of lipid-based vesicles in the airways. The relatively slow evaporation of liposomal AmB allowed prolonged retention of the liposomal AmB in airways. The in vivo tissue distribution study conducted in albino rats of Wistar origin showed that the developed aerosolized liposomal formulations had greater accumulation in the lungs compared to the control, plain uncoated liposomes. This observed higher retention of modified liposomes in alveolar macrophages might be due to their greater and selective affinity for the polysaccharide derivatives on the liposome surface. Further, the polysaccharide coat on liposomes provided protection to the vesicles from destabilization by the Type II alveolar epithelial cells, thus, the duration of high concentration of AmB in lungs was prolonged [71]. The enhanced pulmonary deposition of antifungal through nebulization was demonstrated in a study where liposomal AmB (AmBisome^®^), which was majorly composed of HSPC, CHOL and distearoylphosphatidylglycerol (DSPG), was aerosolized [72]. The nebulized liposomal AmB with the LC Star and Aeroeclipse II nebulizer had the smallest MMAD (<2 µm) and GSD (around 1.9) suggesting uniform particle size and the optimal diameter range of pulmonary delivery. Both the nebulized liposomal AmB showed high FPF < 3.3 µm (>85%) indicating the possibility of delivering aerosol to highly perfused peripheral regions of the lungs. This penetration of the liposomal aerosol to the periphery of the lungs may result in rapid drug availability [73]. A similar study in rats investigating the in vivo aerosol deposition of liposomal AmB demonstrated low systemic exposure of AmB following nebulization of liposomal AmB [74]. Besides, minimal foaming was visually observed during the nebulization experiment of liposomal AmB, which could result in lower deposition of AmB in the oropharynx, enhanced pulmonary deposition and lesser gastrointestinal toxicities of aerosolized AmB [73]. These findings were congruent with a study of intrapulmonary disposition of aerosolized AmB lipid complex in lung transplant recipients, which achieved high concentration of AmB in the epithelial lining fluid with minimal systemic exposure of AmB in the subjects studied [75]. Subsequently, another study was carried out to predict the efficacy and toxicity of pulmonary administration of liposomal AmB [76]. In this study, the liposomal AmB was also prepared from HSPC, CHOL and DSPG. The developed liposomal AmB had a mean diameter of 94 ± 0.1 nm with polydispersity index (PDI) of 0.110 ± 0.020. The highest quantities of AmB recovered were 29.5% in stage 4 and 47.5% in the filter, which correspond to the bronchi and the pulmonary alveolar cells. This indicated that the capacity of liposomal AmB aerosols to reach the deepest part of the lungs where the pulmonary fungal infections usually occur. Although the concentration of AmB in the filter (54 µM) represented was above the minimum inhibitory concentration (MIC) of most of the fungal species (around 1 µM) in the alveolar compartment. It was found that the concentration of AmB delivered through nebulized liposomal AmB might approach the concentration, which could be cytotoxic to the alveolar cells as the alveolar macrophages will ingest the liposomes and release the free drug. Hence, the doses of liposomal AmB delivered through pulmonary route need to be carefully controlled depending on the device used, protocol used and the immune status of the patient [76]. The results of these studies demonstrated the capability of liposomes as nanocarriers to reduce the cytotoxicity of antifungal agents due to retardation of free drug release and enhanced antifungal activity due to enhanced solubility of antifungal agents in liposomes.

Solid lipid nanoparticle and nanostructured lipid carrier approaches to deliver drugs in lungs using nebulization techniques

**Figure 4:**
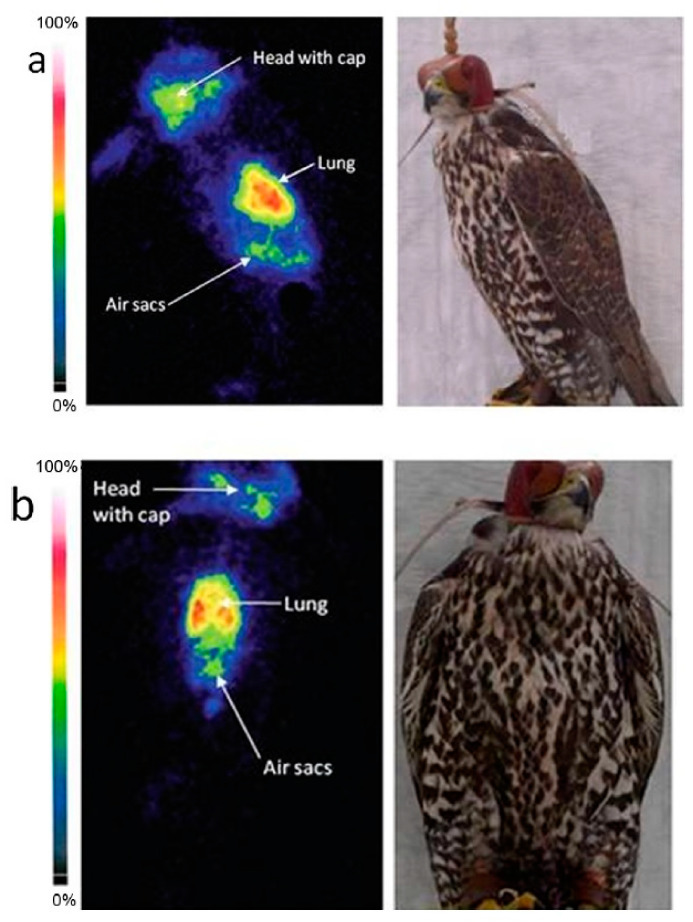
A gamma-camera image of a falcon after inhalation of the developed ITZ-NLC formulation from (**a**) the lateral side and (**b**) from dorsal side [79].

Pardeike and team developed ITZ-loaded NLC formulations for nebulization with solid lipid Precirol ATO 5 and liquid lipid oleic acid (9:1). ITZ was dissolved in the lipid mixture of the developed NLC formulation. The surfactant Eumulgin SLM 20 had the lowest contact angle (79.67 ± 1.16°) due to the reduction of interfacial tension between the lipid matrix and the aqueous phase. The isotonicity of the formulation was also considered by using glycerol 85%, considering that the addition of electrolytes might destabilize the formulation electrostatically. As a result, the developed NLC formulation had enhanced physical stability due to both electrostatic stabilization and steric hindrance of surfactant chains. The stability of the formulation was maintained even after autoclaving. The developed NLC formulation displayed homogeneous particle size distribution (around 200 nm) and a high EE (98.78%). This high EE was due to the low aqueous solubility and high affinity of the lipophilic ITZ to Precirol ATO 5 and oleic acid. Moreover, the crystal order disturbance due to the mixture of liquid lipid and solid lipid produced an imperfect lipid matrix in the crystal lattice structure, which can provide larger space for accommodation of ITZ in the matrix. The developed ITZ-NLC formulation demonstrated burst release profile where 80% of ITZ was released within 5 min. This might be due to the large surface area provided by the NLC in addition with the short diffusion distance for ITZ from the lipid matrix into the dissolution medium as the drug is enriched in the outer region of the NLC. The developed NLC formulation also possessed good storage stability as the particle size, zeta potential and EE do not change significantly over the observation period of 6 months under both room temperature and refrigerated conditions. The developed ITZ-NLC formulation was stable during nebulization as there were no significant changes in particle size and EE of the nebulized formulation indicating absence of particle agglomeration, aggregation, fragmentation of the NLC, which can cause system instability. The ITZ-NLC formulation was predicted to possess increased deposition in all lung regions due to the increased diffusional mobility of the particles with particle size of <500 nm [77]. Autoclaving is a crucial process of sterilising formulations but there is a concern that the process may affect the stability of the developed NLC formulations. Thus, a study had evaluated the effect of autoclaving on the stability of the ITZ-loaded NLC formulation with the same excipients as described in the previous study [77]. Results showed that autoclaving had no influence on the particle size (100–250 nm) and zeta potential of the developed NLC formulation, suggesting unchanged surface properties of the nanoparticles. Hence, the stable NLC formulation in this study may suggest the suitability of the stabilizer and lipid derivatives used remained stable after autoclaving. However, there were multilamellar structures formed from the ITZ-loaded NLC, this induced reorganization of the NLC into unique matrix make it useful in delivery of insoluble drug, ITZ. The changes in structure also caused the NLC formulation to have higher viscosity due to the formation of lamellar network and the formulation was physically stable when stored for 6 months at room temperature as compared to non-autoclaved ITZ-loaded NLC. Thus, the multilamellar structures formed through autoclaving enhanced the storage stability of ITZ-loaded NLC and it was assumed that the lamellar bilayer system formed was similar to biological membranes, which contributes to the high physical stability of the system. This could be due to the enclosure of poorly soluble ITZ in the lamellar layers similar to transmembrane proteins [78]. To have a clear picture on how the developed ITZ-NLC formulation works in vivo, the same group had developed ITZ-NLC formulation for nebulization to treat PA in falcons [79]. The formulation was prepared using the same excipients as the previous study [77]. The developed ITZ-NLC formulation had high EE (99.98%) and zeta potential (−28.7 ± 1.7 mV) indicating good long-term stability due to the avoidance of particle aggregation by electrostatic repulsion between the particles and steric hindrance. Furthermore, the developed NLC formulation was reported to be aerosolized using nebulizer with no physical instabilities detected evident through unchanged particles size of the carrier system during nebulization. The developed NLC formulation had particle size ranging from 100–200 nm, indicating the possibility of the particles to penetrate deep into the respiratory system and deposit there by diffusion. This was evident from the detection of ITZ-loaded NLC in both lung lobes and in the caudal air sacs of the falcon through gamma-scintigraphy imaging acquired after aerosolization and inhalation of the ITZ-NLC formulation (Figure 4). Majority of the radiolabeled ITZ-NLC inhaled were shown to reach the lung and air sacs of the falcon [79].

Other approaches to deliver drugs in lungs using nebulization techniques

Nanoemulsions are isotonic mixtures of drugs, lipids, where the dispersion phase is stabilized by the thin layer of surfactants with droplet diameters ranging from 10 to 500 nm. These structures provide good kinetic stability and high drug solubilizing capacity for lipophilic drugs [80,81]. A study by Nasr et al. developed AmB-lipid nanoemulsions for nebulization with two commercially available isotonic nanoemulsions, Intralipid^®^ 20% and Clinoleic^®^ 20%. Both nanoemulsion formulations were stable as no aggregates and significant change in pH were observed. Both nanoemulsion formulations had high DL efficiency, where Intralipid^®^ nanoemulsion had reported with comparatively higher DL efficiency (87.46 ± 2.21%) as compared to Clinoleic^®^ nanoemulsion (80.76 ± 0.70%). The high DL efficiency of both nanoemulsion formulations might be due to the presence of lecithin in both nanoemulsions, which emulsified and improved the solubility of lipophilic drug. Besides, the presence of polyunsaturated acids (soybean oil in Intralipid^®^ and olive oil in Clinoleic^®^) also improved the incorporation of AmB in the nanoemulsions. Both AmB nanoemulsions had high aerosol and drug outputs (around 90%) due to the presence of oil and surfactant in the nanoemulsions with volume median diameter of 5.00 ± 0.07 µm for AmB Intralipid^®^ and 4.41 ± 0.19 µm for AmB Clinoleic^®^. However, AmB Clinoleic^®^ had higher FPF (80%) than AmB Intralipid^®^ (57%) which could be attributed to the different excipients in each formulation. The corresponding doses of AmB delivered in aerosol droplets smaller than 2.15 µm were 3.3 mg/3 mL for Intralipid^®^ and 3.6 mg/3 mL for Clinoleic^®^ as compared to intravenously administered Fungizone^®^ at a dose of 1 mg/20 mL over 20–30 min. This satisfactory in vitro nebulization performance indicated the possibility of delivery of AmB nanoemulsion formulations as aerosols by nebulization [82]. Another study developed different lipid-based formulations of AmB in the form of lipid micelles, where an AmB lipid-based nanoparticle nebulization formulation was developed with sodium deoxycholate sulfate (SDS). The developed SDS-AmB nebulization formulation had high zeta potential (−40 mV) which might be attributed to prevention of the flocculation or aggregation of the particles due to Van der Waals’ forces. In the antifungal activity assay using *Saccharomyces cerevisiae* (ATCC 9763), the developed SDS-AmB formulation in the form of reconstituted dry powders showed potency of 103% indicating that the lipid did not enhance the transportation of AmB into fungal cells. However, the lipid was important in the dissolution of the poorly water soluble AmB in water. The developed SDS-AmB formulation also exhibited lower MIC and MFC values against *C. albicans* and *C. neoformans* than pure AmB suggesting the stimulation of antifungal activity of AmB by SDS and its importance for the solubility of AmB through formation of lipid micelles. The formulation also displayed good aerodynamic performance with MMAD of 1.74 µm, FPF of around 80% and GSD of 2.0 [83].

Polymeric drug delivery approaches to deliver drugs in lungs using nebulization techniques

Polymeric micelles represent another polymeric drug delivery system approach that has been explored to aid the delivery of drugs. Polymeric micelles are useful in increasing the water solubility of sparingly soluble drugs, stability and the bioavailability of the drugs [84]. Apart from that, their sizes are small, ranging from 10 nm to 200 nm, and they have simple preparation procedures. Polymeric micelles have more advantages than the surfactant micelles as they have a higher loading capacity core to load more drugs, improved stability and longer retention times [85,86]. Polymeric micelles can be obtained from the amphiphilic di-block and tri-block copolymers as well as graft polymers [87]. When these amphiphilic block copolymers are dissolved in an aqueous medium above the critical micellar concentration, amphiphilic block copolymers will self-assemble spontaneously to form spherical core–shell structures [85,88]. Polymeric micelles have both hydrophobic and hydrophilic regions in the core–shell structures [89]. They are gaining popularity in delivery of drugs as the core surrounded by the shell is highly lipid soluble, which is suitable to deliver hydrophobic drugs. Whereas the hydrophilic shell is vital to the in vivo behaviours of the polymeric micelles. The shell provides steric hindrance, inhibit opsonisation and prevent clearance by the reticuloendothelial system (RES), extending the circulation time of the polymeric micelles in the blood [86,87,89]. Polymeric micelles have been investigated to incorporate with antifungal and delivered via nebuliser in several studies.

Due to the lack of hydrophobic regions, chitosan does not have the ability to form micelles on its own. Stearic acid composed of hydrophobic fatty acids chain can be introduced into chitosan to form an amphiphilic copolymer, which can further produce micelles. With this concept, Gilani et al. developed polymeric micelles loaded with AmB with depolymerised chitosan and stearic acid (DCSA-AmB) using a solvent evaporation method. According to the reported results, the DCSA-AmB had the average particle sizes <250 nm. It was reported that higher amount of drug loaded into the micelles will decrease the size of the micelles as the increased hydrophobic interaction among the stearic acid and AmB. This resulted in the formation of a tightly packed micelle core. Apart from that, the particles of DCSA-AmB showed high positively charge zeta potential (44.2–58.5 mV) which reflected the high stability of the formulation due to increase repulsion force and decrease agglomeration. Maximum EE was achieved by the DCSA-AmB, which was 97%. This result was corresponding to the amount of stearic acid that combine to the chitosan as higher amount of stearic acid offers larger hydrophobic area to encapsulate the highly hydrophobic AmB. DCSA-AmB showed higher solubility (355 mg/mL) compared to the free AmB that had intrinsic solubility of smaller than 1 mg/mL. In vitro antifungal activity of DCSA-AmB was compared to the marketed AmB, Fungizone^®^ and nearly similar MICs were obtained in most of the cases. Besides, DCSA-AmB obtained a nebulization efficiency of around 56% and FPF ranging from 40%–50%, which did not differ much from the Fungizone^®^ suggesting the added excipients will not affect its aerosolization performance. Lastly, the encapsulation of drug in DCSA-AmB did not differ much after the nebulization process, which might produce shear force and affect the encapsulation indicating high stability of DCSA-AmB. The results of the study obtained showed high potential of DCSA-AmB for pulmonary delivery of AmB through nebulization [90]. Similar to AmB, the efficacy of triazole is limited by its low water solubility. Approach such as adding high concentration of excipients to improve the solubility of triazole often results to the toxicity. Thus, Moazeni et al. had developed polymeric micelles loaded with ITZ with depolymerised chitosan and stearic acid (DCSA-ITZ) using film hydration to improve the physical properties of ITZ, including solubility [91]. The maximum EE obtained was around 50%, where DCSA-ITZ had the capability to entrap a maximum of 43.2 µg/mL of ITZ which reflected the higher solubility compared to ITZ that has a reported aqueous solubility of 0.00964 mg/mL [91]. DCSA-ITZ had average particle sizes ranging from 120 to 200 nm that were directly proportional to the concentration of added polymer [91]. In terms of FPF, the results fell between 38 to 47%. Whereas, the nebulization efficiency was around 90% [91], which was higher than previous study [90]. In addition, high percentage of drug encapsulated after nebulized showed that DCSA-ITZ remained stable during the nebulization process. Furthermore, an in vitro release study showed that DCSA-ITZ exhibited instantaneous release of 49% of the drug during the first 12 h followed by slowed release for up to 60 h. This fast release might be due to the adsorption of drug molecules at the outer regions of the polymeric micelles. In vitro antifungal activity assay showed that DCSA-ITZ had similar MIC value compared to ITZ that dissolve in dimethyl sulfoxide (ITZ-DMSO). This might be due to the DCSA-ITZ had comparable size distribution to ITZ-DMSO. Thus, the study proved that the enhanced solubility and stable nanocarrier made up of chitosan and stearic acid had high potential to deliver ITZ [91].

Other than polymeric micelles, antifungals loaded into polymeric nanoparticles delivered through nebulization have also been explored by different researchers. Shirkhani et al. prepared nebulized polymethacrylic acid nanoparticles containing AmB (AmB-PMA) for the prevention of pulmonary invasive aspergillosis. Based on the reported results, AmB-PMA had a diameter around 78 nm, indicating it could reach to the terminal bronchioles of the lungs. AmB-PMA had 4 mg/mL solubility, which was much higher than the free AmB (<1 mg/mL). Additionally, it was found that the AmB-PMA remained stable in the condition of 4 °C for 18 months. The nebulized AmB-PMA showed that 300 µg of AmB-PMA able to inhibit the growth of *A. fumigatus* infected BALB/c mice. Apart from that, evaluation of in vivo antifungal activity proved that the nebulization of AmB-PMA for 3 consecutive days in immuno-suppressed BALB/c mice infected with *A. fumigatus* had a fungal burden 99% lower than the immuno-suppressed infected and untreated BALB/c mice [92]. In comparison to the aerosolized Fungizone^®^ formulated by Schmitt et al. for prevention of IPA, the dose of AmB-PMA used was lower, with a higher survival rate in the infected mice after 8 days [92,93]. In addition, it was reported that there was a 90% reduction of tumour necrosis factor (TNF-α) in the mice administered with AmB-PMA as prophylactic suggesting inflammation of lungs can be prevented. This was proved by the microscopic appearance of the infected BALB/c received AmB-PMA had normal lungs while, infected BALB/c mice without receiving AmB-PMA showed blackened lungs [92].

Instead of polymethacrylic acid, polyester is another non-toxic alternative that can be used to formulate polymeric nanoparticles. In this context, Aoun et al. developed an inhaled AmB-loaded polymeric nanoparticles with PLA-grafted with PEG (PEG-g-PLA-AmB) using an oil-in-water emulsion evaporation method for pulmonary delivery. The developed PEG-g-PLA-AmB had spherical shape and the mean hydrodynamic diameters ranging from 150 nm to 200 nm, which allowed them to be nebulized properly and internalized by the invading fungi. PEG-g-PLA-AmB had low zeta potential of around −5.4 mV with loading efficiency of around 25%. In vitro release assay results showed sustain release manner with 25% of drug released rapidly in the first 24 h followed by controlled release over 10 days.

By using a cascade impactor to study the deposition of PEG-g-PLA-AmB in the lungs, nebulized PEG-g-PLA-AmB was seen to deposited mostly at the bronchial level compared to alveolar regions whereas Fungizone^®^ was mainly deposited in the alveolar regions, suggesting PEG-g-PLA-AmB had more favourable deposition profile. In vitro antifungal activity was carried out on the *Candida albicans, Aspergillus fumigatus* and other *Candida* spp. biofilms with the results showing PEG-g-PLA-AmB exhibited higher antifungal activity with a 2- to 3.3-fold decrease of the half-MIC (IC_50_) when compared to free AmB. This result was proved by the uptake of PEG-g-PLA-AmB into fungal cells in which most of the fungal cells had internalized the fluorescent-labelled PEG-g-PLA-AmB after 2 h post-treatment [94]. Improvement of solubility of the antifungal drugs in polymeric micelles through the reviewed articles demonstrated the possibility of reduced antifungal dosage to be delivered through pulmonary route to treat PA. The nanocarrier-based formulation approaches using nebulization platform against PA are summarized in Table 4.

#### 3.1.3. Intravenous Drug Delivery against Fungal Infection

IV drug delivery was one of the common routes of administration due to its importance in unconscious patients, patients with dysphagia and critically ill patients. Drug delivered though IV route of administration can bypass the first-pass effect and reach the blood stream rapidly with 100% bioavailability. In this section, IV formulations of antifungal agents to fungal infections will be discussed in the context of lung-targeted drug delivery of the formulation and modulation of release pattern of the IV formulations [95].

##### Intravenous Nanocarriers against Pulmonary Fungal Infection:

Lipid based delivery for intravenous administration:

Liposomal delivery of intravenous delivery in the treatment against pulmonary fungal infection

Lung-targeted drug delivery can be achieved through modification of the surface charges of liposomes. In this context, an IV formulation of ITZ-loaded liposomes coated with carboxymethyl chitosan (CMC) was developed. The developed ITZ-CMC IV formulation had liposomes’ diameters in nanometer range (349.3 ± 18 nm) and high zeta potential (−35.71 ± 0.62 mV) indicating a good long-term stability. ITZ-CMC also had a high EE (83.4% ± 2.49). The in vitro antifungal test against *C. albicans* showed that the encapsulation of ITZ in the liposomes did not inhibit antifungal activity. ITZ-CMC IV formulation had a higher mean retention time and AUC of ITZ in the in vivo study carried out in the Kunming mice when compared to the commercially available ITZ injection. The prolonged in vivo drug circulation time was due to the coating of liposomal delivery with CMC, which alleviated the problem of limited drug activity of conventional liposomes due to rapid clearance by the RES, which is evident from the reduced drug distribution in liver and spleen following administration of the developed IV formulation. This avoidance of RES might be due to the ability of CMC to increase the repulsion of the particles where the coating produced a big stereospecific blockade, with the formation of a hydration of the liposomal shell surface, which reduced the recognization and uptake of liposomes by RES. Hence, the in vivo and in vitro stability of the IV formulation was enhanced. Besides, the developed IV formulation also shown targeted delivery of the drug in lungs with targeting efficiency of 49.71% and much higher AUC in lung (436.915 µg/hg) than plain ITZ liposomes (222.309 µg/hg) and commercially available ITZ injection (102.995 µg/hg). Although the CMC surface was negatively-charged, the exposure of positively-charged liposome core due to environment changes and physical adsorption facilitated the significant initiative lung targeting while reducing the drug distribution to other tissues, reducing the side effects of ITZ. This was shown through the reduced drug distribution in heart and kidneys by the developed IV formulation where the drug distribution in heart was reduced from 13.54% to 5.96% and 31.14% to 5.32% in the kidneys. This showed that the CMC-coated ITZ liposomes IV formulation has the potential to decrease the risk of cardiac toxicity and nephrotoxicity as compared to the commercially available ITZ injection where the common adjuvant used in the formulation, hydroxypropyl-β-cyclodextrin (HP-β-CD) was associated with nephrotoxicity [96].

Utilization of other manufacturing methods in developing nanotechnology-based formulations was also ventured into. Thus an IV liposomal AmB formulation was prepared with HSPC, DSPG, CHOL using a supercritical carbon dioxide (SCF-CO_2_) fluid method. *N,N*-Dimethylacetamide and ascorbic acid (vit C) were used as the solvent for the IV formulation as this combination had the highest EE (91.5 ± 2.3%). The developed SCF-processed liposomal AmB had an average particle size of 137 nm. This was due to the high stability of AmB with vit C, which acted as antioxidant to prevent autoxidative degradation of AmB in addition to the stabilizing effect of the components in organic solvents attributed to the decreased intramolecular interactions and increased solubility of AmB in acidic conditions. The rapid precipitation of vit C during the SCF-CO_2_ method allowed most of the drug to remain on the surface of lactose. This supposed to be important as lactose not only acts as a carrier but also as a cryoprotectant in the formulations to provide physical stability to the liposomes and high protection against liposomal drug leakage during lyophilization. The liposomal AmB formulation was shown to be stable, as evident through non-significant changes in particle size during four weeks of storage, indicating absence of liposomes aggregation. This enhanced stability may be due to the static repulsion induced by the carbonic acids incorporated into the bilayer membrane. The in vivo pharmacokinetic study results in Sprague-Dawley rats, the SCF-processed liposomal AmB formulation achieved similar overall AUC_(0–24h)_ with that of the commercial liposomal AmB formulation, AmBisome^®^ [97].

Solid lipid nanoparticle and nanostructured lipid carrier for intravenous administration against lung infections

A study by Jung and team developed an AmB-entrapping lipid nanoparticles (LN) IV formulation with 1,2-dipalmitoyl-*sn*-glycero-3-phosphocholine (DPPC), CHOL, 1,2-dipalmitoyl-*sn*-glycero-3-phophate (monosodium salt) (DPPA) and 1,2-distearoyl-*sn*-glycero-3-phospho-ethanolamine-N-[methoxy(polyethylene glycol)-2000] (ammonium slat) (DSPE-mPEG_2000_). The developed formulations (PEG-LN AmB) had smaller mean particle size (< 100 nm) and high zeta potential (−41.3 ± 5 mV to −50.4 ± 5 mV) which indicated good long-term stability. The smaller particle size and high zeta potential were due to the presence of anionic lipid, DPPA and DSPE, which induced strong repulsive forces between the LNs. Besides, DPPC and DPPA had strong hydrophobic interaction with AmB, which resulted in the high EE of the PEG-LN AmB formulation in addition to the strong interactions of AmB with CHOL. Pharmacokinetics study in Sprague-Dawley rats suggested prolonged circulation half-life of PEG-LN AmB with half-life of 5.591 h as compared with Fungizone^®^ (3.825 h) and AmBisome^®^ (3.673 h). PEG-LN AmB demonstrated an almost 2-fold higher AUC_0–24h_ (19.448 µg.h/mL) than AmBisome^®^ (10.204 µg.h/mL) and 19 times higher AUC_0–24h_ than Fungizone^®^ (1.084 µg.h/mL). This prolonged half-life in the bloodstream was due to the formation of hydrodynamic layer on the LNs by DSPE-mPEG_2000_. which might inhibit RES uptake and opsonization of the LNs. The in vitro antifungal activity of the PEG-LN AmB against *C. albicans* and *A. fumigatus* was better than Fungizone^®^ and AmBisome^®^ where the MIC of PEG-LN AmB against *C. albicans* and *A. fumigatus* were 0.125 µg/mL and 0.25 µg/mL when compared to Fungizone (0.5 µg/mL, 1 µg/mL) and AmBisome^®^ (0.25 µg/mL, 0.25 µg/mL). This might be due to the higher affinity of AmB incorporated into the LNs to ergosterol in fungal cell membrane than to CHOL in LNs. The in vivo antifungal efficacy of PEG-LN AmB tested in ICR mice was superior than AmBisome^®^ with higher mean survival times which might be attributed to the effect of PEG molecules on pharmacokinetic and toxicological behaviour of AmB [98].

To overcome the limitations associated with SLN, NLC, which is the second generation SLN was utilized in the delivery of antifungal agents. In a study, ITZ-loaded NLC IV formulations were developed with tristearin as solid lipid, triolein (TO) as liquid lipid, Tween 80, egg yolk l-a-phosphatidylcholine (eggPC) and DSPE-mPEG_2000_. The developed NLC IV formulations have particle size in nanometer range (192.7 ± 14.4 nm to 241.9 ± 13.5 nm) and high incorporation efficiency (83.0 ± 1.3% to 95.2 ± 1.7%). The high solid lipid concentration serves as immobilization capacity for incorporation of drug into the lipid core while the liquid lipid weakened the crystallinity of the lipid core during lyophilization. The in vitro release study suggested that the release rate of ITZ from the NLC formulations could be controlled by modulation of the amount of liquid lipid in lipid core. This is due to the less mobility of lipid core as compared to liquid-solid LN. As proportion of liquid lipid increases in the formulations, the cumulative drug release of the formulations increased which could be due to faster drug release induced by the weak crystallinity of liquid-solid LN. The pharmacokinetics study in male Sprague-Dawley rats suggested sustained release of ITZ from the NLC formulation with less amount of liquid lipid as LN with high amount of liquid lipid will be degraded rapidly by enzymes in the blood circulation and were cleared in the blood faster. The sustained release of ITZ-NLC was due to higher solid lipid proportion in the formulation as well as the presence of steric stabilizer, DSPE-mPEG_2000_ used in the formulation. In addition to prolonging of the circulation time of NLC in bloodstream, reduction of gel formation during preparation and storage due to steric hindrance induced by DSPE-mPEG_2000_ also increased the stability of the formulation [99]. To further improve the payload of the NLC formulation, ITZ-loaded NLC IV formulation with tristearin, TO, Tween 80, eggPC and DSPE-mPEG_2000_ using hot high-pressure homogenization method has developed with a drug high payload. The ITZ-loaded NLC formulation has high incorporation efficiency (89.6%), loading capacity (18.3%) as well as particle size of 285.9±14.8 nm and PDI of 0.195 ± 0.015. The high DL capacity was due to the formation of disordered of crystal lattice by the addition of small amount of liquid lipid in lipid core, which created larger space for accommodation of ITZ. Besides, addition of liquid lipid into the lipid core also slows down the recrystallization of solid lipid. This reduced crystallinity in lipid core results in reduced drug expulsion during storage [100]. The results of the in vitro dissolution study was in agreement with the study by Kim et al., where the dissolution rate of ITZ could be controlled by modulating the amount of liquid lipid in the formulation [99]. High ITZ deposition in the liquid lipid-enriched outer layer could explain the initial burst release of ITZ while the sustained release of ITZ could be due to the weak crystallinity and high mobility of lipid core in the presence of liquid lipid, where the release rate of ITZ was proportional to the amount of liquid lipid, TO in lipid core. Prolonged circulation time in bloodstream for the high payload ITZ-loaded NLC IV formulation as compared to Sporanox^®^ IV was evident in male Sprague-Dawley rats, whoch might be due to the presence of PEG as stearic stabilizer preventing the rapid clearance of NLC by RES through avoidance of protein adsorption and subsequent opsonization [100].

Other intravenous approaches against lung infections

The particle size of an IV formulation may influence its lung-targeted drug delivery. An IV formulation of AmB was prepared with sodium deoxycholate (DCH) (AmB-DCH), which had a mean particle size of 404.9 ± 1.7 nm. Based on the in vivo drug distribution study in Oncins France 1 male mice, the AmB-DCH formulation has significantly higher drug concentration in lungs as compared to liposomal AmB formulation (9.173 ± 0.498 µg/g versus 2.527 ± 0.386 µg/g) while the drug concentration in kidney of AmB-DCH was 15-fold lower than the liposomal AmB formulation. This suggested better lung-targeted delivery of drug and furthermore, less nephrotoxicity of the AmB-DCH, which might be due to the presence of AmB poly-aggregated form, which is a small size micellar system induced by the surfactant action of DCH and stabilized by the ionic interaction between DCH and AmB. The AmB poly-aggregated form with higher particle size (404.9 ± 1.7 nm) showed greater lung distribution as compared to commercial liposomal AmB (around 100 nm) which showed high renal distribution suggesting influence of particle size on the distribution of AmB to different organs. The higher particle size of AmB-DCH allow faster opsonization of the drug by alveolar macrophages, resulting in higher drug distribution in lungs which would be adequate to treat aspergillosis (MIC 0.5–8 µg/mL) [101]

Polymeric drug delivery system for intravenous treatment of lung infections

Antifungals incorporated in polymeric nanocarriers for IV route administration have also been explored by researchers worldwide. Owing to the biocompatible and biodegradable properties in drug delivery, many researchers have developed polymeric nanoparticles by using a diblock copolymer, namely D-α-tocopheryl polyethylene glycol 1000 succinate-β-poly(ε-caprolactone-ran- glycolide) (PLGA-TPGS). Tang et al. had developed AmB loaded PLGA-TPGS nanoparticles (AmB-NP) using a modified nanoprecipitation method to overcome the limitations of AmB in the treatment of pulmonary fungal infections. The AmB-NP had a mean hydrodynamic diameter between 120 nm to 130 nm, which was claimed effective in cellular uptake and internalization. The PDI of AmB-NP was around 0.2 indicating uniform particles size distribution [102,103]. DL and EE of AmB-NP were 10% and 85%, respectively. The zeta potential of AmB-NP was around −18 mV to −19 mV, which did not hit the minimum desired zeta potential of ±20 mV. However, the average size and size distribution of AmB-NP remained constant for 5 months in in phosphate buffer solution, which reflected high stability and redispersion ability. AmB-NP showed biphasic release pattern during in vitro release study with fast released of 58.3% of drug initially during the first 12 days followed by sustain release of 8.1% of drug over 18 days. The initial burst was due to the drug released from the surface of particles.

**Figure 5:**
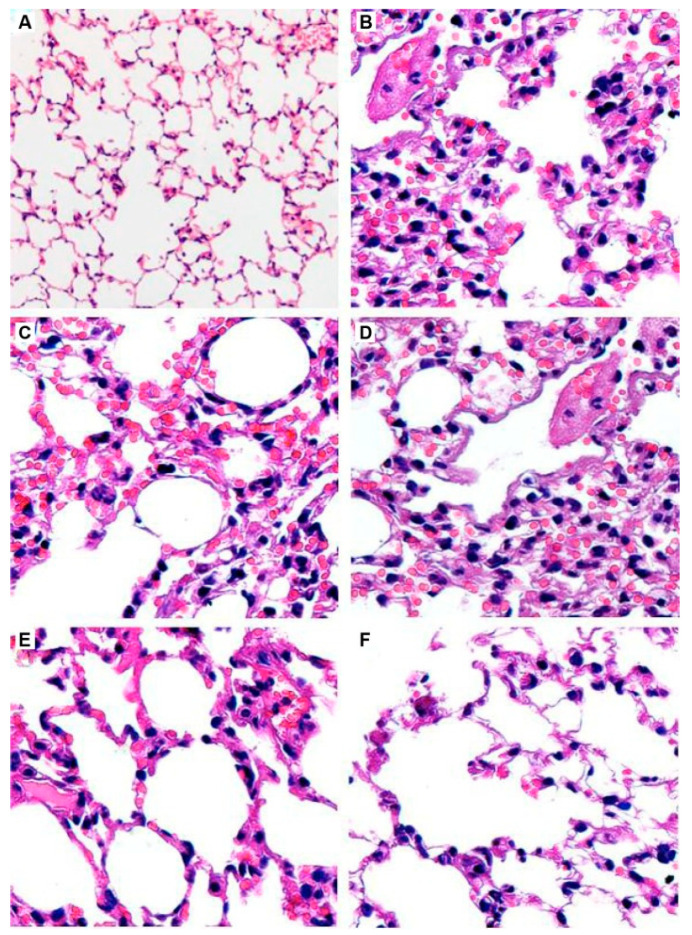
Section (**A**) shows normal lung tissues without any infection. Section (**B**) shows interstitial oedema in the lung tissues of *C. albicans* infected mice with yeast-like organisms after staining with haematoxylin and Periodic acid–Schiff. Section (**C**) and (**D**) showed the lung tissues of *C. albicans* infected mice after receiving phosphate-buffered saline and empty PLGA-TPGS NP which had comparable observation to the section (**B**). Section (**E**) and (**F**) shows lung tissues of *C. albicans* infected mice after treated with free AmB and AmB-NP respectively. Both lung tissues showed improvement compared to the untreated lung tissues; improvement after treatment of AmB-NP is more obvious [102].

Afterward, slow diffusion and particle degradation accounted for the slow release. In vitro antifungal activity measurments showed that AmB-NP had the same MIC value compared to the free AmB. However, less than 50% of drug was released from the nanoparticles initially, which suggested a higher antifungal activity of AmB-NP. Interestingly, the BALB/c mice infected with *C. albicans* which were treated with AmB-NP intravenously had lower fungal burden and higher survival rates compared to the *C. albicans-*infected BALB/c mice treated with free AmB. In addition, the authors found an improvement on the lung lesions of the *C. albicans* infected mice that treated with AmB-NP compared to the infected and untreated BALB/c mice as well as the *C. albicans-*infected BALB/c mice treated with free AmB (Figure 5). In short, AmB-NP is dominant over free AmB [102].

In another study, Tang et al. developed AmB-NP using a modified double emulsion method and evaluated its fungistatic and fungicidal effects. The hydrodynamic diameter of AmB-NP (118 nm to 127 nm) and PLGA-loaded AmB nanoparticles (PLGA-AmB) (107 nm to 117 nm) were comparable. In terms of DL and EE, AmB-NP were in accordance with the previous study. The DL and EE of PLGA-AmB was slightly lower than the AmB-NP reflecting higher binding affinity of lipid soluble drug to the PLGA-TPGS nanoparticles. However, PLGA-AmB had a slightly higher zeta potential of around −23.6 mV compared to the AmB-NP with zeta potential of around −19.5 mV. In vitro release studies showed that the AmB-NP had a biphasic release profile with a initial release of 47.38% of the drug in 5 days followed by a sustained released for 22 days. Both PLGA-AmB and AmB-NP had the similar MIC value against *Candida glabrata* in the in vitro assay. Cellular uptake assays revealed that coumarin-labelled AMB-NPs were closely located around *C. glabrata,* showing high affinity to the fungal cells which results in its high antifungal activity. Further, PLGA-AmB and AmB-NP had higher reduction of fungal burden on the *C. glabrata*-infected BALB/c mice compared to the free AmB, which might be due to the higher stability of the nanoparticles. However, AmB-NP showed higher efficacy than the PLGA-AmB which shown by its higher survival rate on the *C. glabrata*-infected BALB/c mice. Furthermore, lung histological analysis evidenced a reduction of pulmonary interstitial oedema in *C. glabrata*-infected BALB/c mice treated with PLGA-AmB and free AmB but no detectable lesions and oedema found in the lung tissues of the *C. glabrata*-infected BALB/c mice after treatment with AmB-NP. This study reinforced the previous study results showing that AmB-NP is a potential alternative for the treatment of pulmonary fungal infections [104]. In addition to PLGA-TPGS nanoparticles, another research team incorporated PLGA with dimercaptosuccinic acid (DMSA) to form AmB loaded with PLGA and DMSA polymeric nanoparticles (Nano-D-AmB). Based on the results obtained, 6 mg/kg every 3 days of Nano-D-AmB (equivalent to 2.7 mg/kg of pure AmB) showed comparable antifungal activity to the desoxycholate AMB (D-AmB) with dose 2 mg/kg daily (equivalent to 0.9 mg/kg of pure AmB) following injection into *Paracoccidioides brasiliensis* infected mice which reflecting lower dosing frequency, which might be due to the sustained released of AmB from Nano-D-AmB adequate for three doses in one injection. Weight loss is one of the clinical parameters that reflects a health problem. Infected *P. brasiliensis* mice treated with D-AmB showed a 12.4% weight reduction after 30 days of administration, whereas, the *P. brasiliensis*-infected mice that were treated with Nano-D-AmB did not showed a significant weight reduction after 30 days of administration and a total 9.2% reduction of weight only occurred after 60 days post-administration suggesting delayed adverse effects [105]. Further, Souza et al. investigated the pharmacokinetics, physicochemical characteristics, antifungal activity and biocompatibility of Nano-D-AmB. The particles of Nano-D-AmB were spherical, with a size distribution ranging from 341 nm to 398 nm over 21 days. The variation in the particle sizes might be caused by the aggregation of the particles. The formation of aggregates might be due to the particles of Nano-D-AmB had zeta potential of −15 mV at the first day and approximately −21 mV at day 7, 14 and 21 which is lower than the desired zeta potential (±30 mV). An in vitro release study showed that the Nano-D-AmB released the drug in a biphasic manner which was characterized by an instantaneous release of 30.5% of the drug load in 24 h and followed by sustained released over 2 days. The release pattern might be due to initial diffusion followed by both diffusion and degradation of the polymer. In evaluating the pharmacokinetic parameters, the concentration of AmB in the lungs released from both Nano-D-AmB and D-AmB did not differ. To assess the biodistribution of Nano-D-AmB, 99mTc was used to label the nanoparticles and the results showed that high concentrations of Nano-D-AmB accumulated in lungs, liver and spleen following IV route of administration than the 99mTc-DMSA. In vivo antifungal activity assay showed that Nano-D-AmB at a dose of 4.5 mg/kg/3 days produced a similar reduction of the fungal burden compared to the D-AmB with 6 mg/kg/3 days doses. Surprisingly, it was observed that the *P. brasiliensis*-infected BALB/c mice treated with Nano-D-AmB presented high levels of interleukin-12 suggesting an immunomodulation effect of Nano-D-AmB which might benefit the recipients. Together with the previous results, Nano-D-AmB might be an alternative for Anforicin B^®^ which is the marketed product version of D-AmB [106].

Since the focus had been directed towards triazoles as the treatment of pulmonary fungal infections, Cunha-Azevedo et al. recently developed another PLGA-DMSA nanoparticle formulation containing ITZ (Nano-D-ITZ). In comparison to oral free ITZ, IV Nano-D-ITZ showed a higher concentration of ITZ in the lungs, which might be due to the targeting effect of DMSA and entrapment of nanoparticles in the first encountered capillary bed of lungs after IV administration. In addition, ITZ was also found in the spleen and liver of the experimental mice but undetected in the plasma following administration of Nano-D-ITZ. ITZ in spleen was mainly due to the opsonization of nanoparticles, which was then identified and cleared by the phagocytes and tissue macrophage from the blood, whereas, metabolism of ITZ into a huge number of metabolites in the liver by CYP3A4 might be responsible for the concentration of ITZ in the liver. In evaluating the antifungal activity, *P. brasiliensis*-infected BALB/c mice treated with 60 µg three times daily of Nano-D-ITZ over 30 days showed lesser lesions and fungal burden compared to the treatment with 1 mg daily of free ITZ indicating higher antifungal activity of Nano-D-ITZ. The developed formulation with lower dose and dose frequency provided higher efficacy than the free drug suggesting another alternative to replace the free drug [107]. Nanocarrier based different approaches via intravenous administration for the treatment of PAS are summarized in Table 5.

## 4. Expert Opinion

The delivery of antifungal agents to treat pulmonary fungal infections through pulmonary route has been investigated for the advantages mentioned in this review. Additionally, the use of nanocarriers in pulmonary delivery of antifungal agents has shown impressive potential but the full potential of nanocarriers has yet to be exploited. From the available literature, micronized drugs show enhanced dissolution profiles and antifungal activity. This is beneficial for the delivery of poorly water-soluble antifungal agents as micronized drug formulations provide the possibility of using a reduced dose of antifungal, which subsequently reduces the systemic exposure of the drug, leading to reduced toxicity of the antifungal agents. Besides, the microstructured crystalline formulation of antifungal agents for pulmonary route of administration could provide prolonged retention of the entrapped drug in the lungs, allowing local action of the drug while avoiding extensive systemic exposure of the antifungal agents, thereby reducing associated toxicities.

The formulations delivered through pulmonary routes focus on the aerosolization properties of the formulation to estimate the in vitro deposition of drug in the lungs. Good aerosolization properties of the formulations developed suggest deep lung penetration of the antifungal agents, which would be beneficial in the treatment of pulmonary fungal infections where most fungi colonize the deep lungs. With comparable aerosolization properties, DPI formulations with lipid-based nanocarriers possess advantages over the DPI formulations with only course carriers in that they offer the opportunity of modulated release of the drug. Depending on the quantity and type of the phospholipid used in the formulations, the DPI formulations with lipid-based nanocarriers can achieve burst release profiles or sustained release profiles. The different release profiles of drugs depend on the interaction of the antifungal agents with the lipid components of the lipid-based nanocarriers, which could result in rapid expulsion of the encapsulated drug or a slower release of free drug from the lipid barriers. From the literatures reviewed, reduced cytotoxicity of the antifungal agents could be achieved through incorporation of antifungal agents into lipid-based nanocarriers. Besides, the use of lipid-based nanocarriers also provided the possibility of enhanced targeting of the antifungal agents through incorporation of targeting ligand as compared to DPI formulations with only course carrier.

In addition to lipid-based drug delivery systems, polymeric drug delivery systems in DPI formulation have also being studied widely to enable efficienct delivery of antifungal agents and to overcome the drawbacks of the conventional antifungal for the treatment of pulmonary fungal infections. Based on the reported research, it could be said that the polymeric nanoparticles containing antifungals in DPI formulation possess good aerosolization properties, which increases their efficacy in treating pulmonary fungal infections. Sustained release is a major advantage of the polymeric drug delivery systems which was shown in most of the polymeric formulation developed by researchers that may also enhance the efficacy of the therapy and patient compliance due to the reduction in the frequency of dosing when compared to conventional therapy. It can also be pointed that polymeric drug delivery can improve the solubility of hydrophobic antifungals as AmB and triazoles that have the limitations of low solubility that hinder their effectiveness. Overall, microcarriers and nanocarriers are promising approaches that aid in the delivery of antifungals to the lungs, which could overcome the limitation of conventional therapy in the treatment of pulmonary fungal infections.

## 5. Safety Concerns of Dry Powder Inhaler Formulations against Pulmonary Aspergillosis

The suitability of pulmonary drug delivery using nanocarriers is demonstrated by their enhanced efficacy, stability, and most importantly, their improved safety profile. These improved safety profiles of pulmonary drug delivery formulations of antifungal agents using nanocarriers are illustrated through the use of ideal excipients as well as reduced cytotoxicity of the developed formulations. The use of ideal excipients to enhance the safety of the formulation developed was demonstrated in the study by Beinborn et al. where they judiciously used PVP K25 as the polymeric stabilizer in the formulation [38]. The reasons included, PVP K25 in an FDA-approved product [108], safety results in preclinical studies (no signs of cytotoxicity against cultured human bronchial epithelial cells (Calu-3) and human alveolar epithelial cells (A549)) for pharmaceutical formulations containing PVP [109]. Besides, the utilization of safer excipients was also demonstrated by Shah et al. where ethyl acetate and ethanol were used in combination as the organic solvents to develop LDPI AmB formulations [50]. Both ethyl acetate and ethanol are organic solvents with low toxicity potential [110]. Apart from that, PLA used in formulating the VLM by Arora et al. has shown non-cytotoxicity to the Calu-3 cell line as both VLM and free VRZ did not significantly lower the cell viability [40]. In addition, two different VRZ microparticle formulations showed similar results in cellular viability tests [41,42]. During further toxicity evaluations of the VRZ microparticle formulations, around a 1.6 fold-lower plasma exposure was observed after administration of a VRZ microparticle formulation into Balb/c mice through the inhalation route when compared to the intravenous route reflecting systemic exposure of VRZ was reduced, which may also decrease the associated adverse effects of VRZ [42].

The enhanced safety profile of the pulmonary formulations with nanocarriers was also demonstrated through reduced cytotoxicity against cultured cell lines. A study by Vyas et al. demonstrated lower toxicity of aerosolized liposomal AmB formulation to human RBC in the hemolysis assay. This reduced hematotoxicity of the liposomal formulation can be attributed to the more stable and compact configuration of bilayers and intercalation of AmB in the lipid bilayers [71]. This finding was consistent with the study by Fauvel et al., which showed better tolerance of liposomal AmB in a cytotoxicity assay with human alveolar basal epithelial cell line (A549), where only the highest concentration of liposomal AmB (200 µm) showed some level of toxicity (viability reduced to 87%) [76]. This reduced cytotoxicity of liposomal formulation of antifungal agents could be suggested to be due to a slower release of drug from liposomes due to retarded direct contact of AmB with RBC by lipid bilayers and interaction between AmB with phospholipids and cholesterol [97].

Beside liposomes, NLCs, lipid micelles and SLNs have also demonstrated enhanced safety profiles through reduced cytotoxicity. For instance, the NLC formulation developed by Pardeike et al. showed no signs of cytotoxicity on A549 cells as evident through undetected effect on cell mitochondria and on the membrane integrity of A549 cells at therapeutically used concentrations. This indicated good tolerability of ITZ-loaded NLC, which could be due to the utilization of well tolerated lipids (Precirol ATO 5 and oleic acid) and an optimized concentration of surfactant (Eumulgin SML 20) in developing the NLC formulation [79]. Another example of reduced cytotoxicity was shown by the lipid micelles formulation developed by Gangadhar et al., where significantly lower hemolysis effects on human red blood cells were produced by the SDS-AmB formulation in the in vitro hemolysis assay due to the stabilizing effect of binding between the AmB and SDS [83]. Micelle stabilization through interaction of sulfate ions to AmB slowed down the release of AmB, inhibiting the formation of dimeric forms which lead to haemolysis [111]. Besides, the developed SDS-AmB formulation showed very low cytotoxicity in A549, Calu-3 and alveolar macrophage cell lines (Ams NR8383) as compared to pure AmB. This reduced toxicity in these three cell lines is also due to the ability of SDS to form stable micelles which slow down the release of free AmB [83]. Finally yet importantly, SLNs also demonstrated reduced cytotoxicity in a study by Jung and team. The PEG-LN AmB formulation showed less cytotoxicity in a human kidney cell line than the commercially available formulations, Fungizone^®^ and AmBisome^®^. This was due to the slower release of drug from the stronger barrier provided by the lipid matrix of LN, which suggested that the LN formulation of AmB could reduce the nephrotoxicity more effectively. Lower hematotoxicity of the LN formulation of AmB towards Sprague-Dawley rat RBC suggested lowered direct contact of AmB to RBC due to lipid matrix of LN and slower release of drug due to interaction of AmB with phospholipids and cholesterol [98].

**Figure 6:**
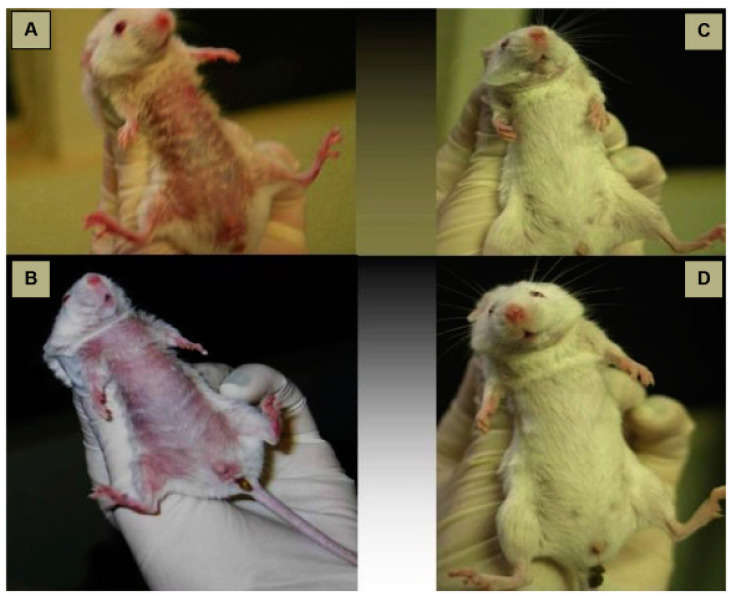
*P. brasiliensis*-infected BALB/c mice in sections (**A**) and (**B**) presented alopecia after the treatment of free ITZ whereas *P. brasiliensis*-infected BALB/c mice in sections (**C**) and (**D**) after the treatment of Nano-D-ITZ presented normal pelage [107].

In term of safety of polymeric drug delivery system, several researchers have performed toxicity evaluations on various cell lines or animal models. Shirkhani et al. found that below 500 µg/mL AmB-PMA was non-toxic to the A549 cells and monocyte derived-macrophages. This showed that AmB-PMA had advantages over Fungizone^®^ as the lytic and cytotoxic effects of deoxycholic acid of Fungizone^®^ can be prevented [92]. Besides, another polymeric nanoparticle formulation, namely PEG-g-PLA-AmB, prepared by Aoun et al. demonstrated a non-cytotoxic nature to pulmonary cell lines including A549 and Calu-3 cell lines for 48 h up to 10 mg of the formulation per mL [94]. Apart from that, AmB-NP prepared by Tang et al. showed decreased toxicity compared to the free AmB, whereby the concentrations of both AmB-NP and free AmB that led to the lysis of all the red blood cells in vitro were 500 µg/mL and 60 µg/mL, respectively. Interestingly, the unloaded nanoparticles showed no haemolytic ability to red blood cells when the concentration was below 1.0 mg/mL. Moreover, an in vivo toxicity assay was performed on mice receiving the treatment intravenously, and the results showed that the lower concentration of AmB-NP than that of free AmB resulted in 50% of mortality in the mice which again proved that AmB-NP had lower toxicity and higher efficacy [102]. In a study by Amaral et al., although 6 mg/kg every 3 days of Nano-D-AmB was expected to cause severe toxicity to the animals as the recommended dose of AmB is 1 mg/kg/day, renal and hepatic biochemical parameters including blood urea nitrogen, creatinine, glutamic oxaloacetic transaminase and glutamic pyruvic transaminase showed normal after the administration of Nano-D-AmB. The absence of toxicity from Nano-D-AmB is most likely due to the site-targeting effect offered by the DMSA and low blood circulation of pure AmB owing to the sustained release property of Nano-D-AmB. Apart from that, no genotoxicity and cytotoxicity in the bone marrow cells were found on Nano-D-AmB after performing a micronucleus assay. The absence of toxicity might be due to the sustained release property and low accumulation of AmB due to the complexation of polymeric nanoparticles [105]. Nano-D-AmB was further tested by another researcher in order to evaluate the in vitro biosafety, where the results showed that Nano-D-AmB induced lesser haemolysis level even in lower concentration when compared to Anforicin B^®^, another commercial AmB product used in Brazil. Apart from that Nano-D-AmB did not show significant viability of peritoneal macrophages which also proves the safety of the formulation, whereas the viability of peritoneal macrophages was reduced after incubation with Anforicin B^®^ [106]. In another study by Cunha-Azevedo et al., it was observed that *P. brasiliensis*-infected BALB/c mice receiving free ITZ presented alopecia (Figure 6), which was not observed in the group treated with Nano-D-ITZ, suggesting diminishment of the adverse effects of ITZ. This might be due to the sustained released of ITZ from Nano-D-ITZ, lower concentration injected and lesser dosing rate [107].

Based on the reviewed literature, it was demonstrated that the antifungal formulations using lipid-based and polymeric nanocarriers have enhanced safety profiles as compared to commercially available antifungal formulations. This suggests the feasibility of the investigated nanocarriers in the delivery of antifungal agents as an alternative for commercially available formulations. However, clinical studies in human subjects are required to further confirm the safety profile, interactions and pharmacokinetic profile of the developed formulations with lipid-based nanocarriers.

## 6. Clinical and Regulatory Aspects Related to Dry Powder Inhalers against Pulmonary Aspergillosis

The pulmonary delivery of antifungal agents with DPI formulations are promising and have progressed to the conductance of clinical trials. Currently, corticosteroids are used in the treatment of patients with ABPA as there is no approved treatment for this condition. The use of corticosteroids can alleviate the inflammatory response to *Aspergillus* spp. colonizing the lungs of the patients while some patients also require systemic treatment with antifungal agents, such as ITZ. Despite their effectiveness in the treatment of ABPA in some patients, corticosteroids and systemic antifungals are associated with some serious side effects.

PUR1900 (Pulmazole^TM^) is a dry powder inhaled formulation of ITZ currently under development by PULMATRIX (Lexington, MA, USA), a clinical stage biopharmaceutical company. It is an investigational product which is intended for the treatment of ABPA to curb the limitation of oral antifungals and to potentially reduce the steroid burden [112]. The product has undergone Phase 1 clinical trials while the Phase 2 clinical trial was halted due to the COVID-19 pandemic [113,114].

## 7. Conclusions

The increasing incidence of pulmonary fungal infections, particularly in immunocompromised patients, requires highly efficient delivery of antifungals into the lungs to prevent and eradicate the fungal cells. The development of novel approaches that target the lungs are needed to compensate the limitations of the conventional therapy of pulmonary fungal infections. Lipid- and polymer-based nanotechnology in cooperation with antifungal formulatiosn have shown positive results regarding enhanced pharmacokinetic and antifungal activity. Thus, targeted delivery by administering these nano-formulations via the pulmonary route is the promising option for the treatment of pulmonary fungal infections. However, future studies and more well-designed clinical trials are needed to ensure the suitability of the nano-formulations for effective and safe delivery of antifungals, as well as to elucidate their associated risks.

## Figures and Tables

**Table 1:** Micronized drugs administered as dry powders for lung delivery.

Objective	Polymer Used	Drug	Manufacturing Method	Cell Line/Animal Model	Outcome of the Research	Source
To assess the potential of AmB incorporated in SCC, cholesteryl palmityl carbonate and dicholesteryl carbonate for use in dry powder aerosol.	SCC, cholesteryl palmityl carbonate, dicholesteryl carbonate	AmB	Solvent evaporation	*C. neoformans* (ATCC 90113) and *C. albicans* (ATCC 90028)	AmB-SCC MMAD (3.8 ± 0.7 µm) ED (87.9 ± 1.3%) FPF (38.0 ± 1.3%) GSD (3.02 ± 0.62) AmB-CCE DPI has MIC and MFC 2–4 times higher than pure AmB.	[34]
To evaluate one DPI formulation strategy consisting of the formation of a poorly water-soluble drug based SDs produced by spray-drying process to achieve a high lung deposition, an improved solubility and dissolution profile and an acceptable safety profile in regards with excipients used.	TPGS 1000, mannitol	ITZ	Spray drying	NA	MMAD (1.61 ± 0.05 µm) ED (53 ± 2%) FPF (47 ± 2%) ITZ-SDs have 4 to 6-fold greater saturation levels than unformulated ITZ. ITZ-SD has better aerosolization properties compared to bulk ITZ.	[35]
To evaluate the dissolution and aerosolization of ITZ-based SDs with mannitol and the hydrogenated soy-lecithin.	Hydrogenated soy-lecithin, mannitol	ITZ	Spray drying	NA	Aerodynamic diameter range (2.82–4.46 µm) ED (84.9 ± 5.3%) FPF (66.4 ± 3.6%) ITZ-SD2 has improved dissolution rate than raw crystalline ITZ. ITZ-SD2 showed possible modulated release properties.	[36]
To evaluate the influence of phospholipids on the pharmacokinetic profile in vivo after pulmonary deposition of ITZ DPI SD.	Mannitol, Phospholipon 90 H^®^	ITZ	Spray drying	Male outbred ICR mice	MMAD (around 2 µm) d (0.5) of between 3.9 and 5.59 µm ITZ-SD with phospholipid has significantly higher dissolution rate than ITZ-SD without phospholipid. ITZ-SD without phospholipid achieved greater ITZ lung retention and total exposure of ITZ to lungs than ITZ-SD with phospholipid. ITZ-SD without phospholipid achieved higher AUC_0–24h_ than ITZ-SD with phospholipid	[37]
To evaluate the physicochemical and aerodynamic properties of the VRZ formulations produced by TFF in vitro to determine how these properties affect the pharmacokinetic profile and systemic bioavailability after dry powder insufflation in mice.	1,4-dioxane, PVP K25	VRZ	TFF	Male outbred ICR mice	TFF-VRZ MMAD (4.2 µm) ED (81.2%) FPF (37.8%) GSD (2.4) TFF-VRZ-PVP K25 MMAD (5.2 µm) ED (92.5%) FPF (32.4%) GSD (2.6) TFF-VRZ-PVP K25 has 1.3 times higher area under the dissolution curve than TFF-VRZ. TFF-VRZ has higher lung bioavailability than TFF-VRZ-PVP K25. TFF-VRZ has prolonged lung retention than TFF-VRZ-PVP K25.	[38]
To prepare inhaled VRZ dry powder formulation with sustain release property.	PLA	VRZ	Spray drying method	Calu-3 cells	MMAD was around 3.68 µm, GSD was 2.13 and FPF was approximately 43.56%. Emitted FPF of VLM is 2 folds higher than VLC. Sustain released of drug for 48 h.	[40]
To prepare pulmonary delivery of large porous VRZ microparticle dry powder formulation.	PLA, PLGA 752H, PLGA 502, ammonium bicarbonate	VRZ	Double emulsion method	Lung adenocarcinoma (A549) cells, murine macrophage cell line (RAW 264.7)	MMAD was around 2.85 µm and FPF was around 27.3% Sustain released of drug over 7 days Able to escape uptake from macrophage	[41]
To prepare and characterize inhalable VRZ dry powder formulation.	Leucine	VRZ	Spray drying method	Calu-3 cell line (HTB-55), BALB/c mice	MMAD was around 3.79 µm and FPF was around 60% Exposure of VRZ in the lungs were 15 folds higher via inhalation compared to intravenous delivery	[42]
To prepare micronized cocrystal dry powder formulations of ITZ to improve pulmonary absorption.	Succinic acid, malic acid, fumaric acid, tartaric acid, D-mannitol	ITZ	Jet milling	Sprague-Dawley rats	Mean diameter was 2.9 µm. Dissolution rate of ITZ-SA is 10 times and 5 times higher than pure ITZ and other cocrystal respectively. The micronized ITZ-SA shows 2 folds and 24 folds higher of AUC0–8h compare to amorphous ITZ and crystalline ITZ respectively. Bioavailability of ITZ-SA was 10.9% which was higher than amorphous ITZ (5.6%) and crystalline ITZ (0–5%).	[43]
To develop ITZ NanoCluster formulation via wet milling method.	Ethanol	ITZ	Anti-solvent precipitation, wet milling	NA	MMAD (1.2 ± 0.1 µm) ED (80.3 ± 10.7%) FPF (91.8 ± 1.2%) GSD (2.0 ± 0.1) ITZ NanoCluster formulations prepared by wet-milling technique showed better aerosol performance than micronized ITZ.	[44]

**Table 2:** Nanocarriers for lung delivery through DPIs.

Objective	Type of Nanocarrier	Polymer Used	Drug	Method of Preparation	Cell Line/Animal Model	Outcome	Source
To prepare and optimize liposomal AmB DPI formulation for treatment of invasive lung fungal infection.	Liposome	HSPC, CHOL, α-tocopherol, SPG-3, SA	AmB	Reverse phase evaporation technique, spray freeze drying	NA	Angle of repose (28.3 ± 0.6°) Dispersibility index (20.8 ± 1.0) Compressibility index (23.5 ± 1.8) Effective index (44.8 ± 1.6) FPF (22.5 ± 2.2%) The LDPI AmB has high drug EE (80%). The LDPI AmB demonstrated delivery of liposomally entrapped AmB from tracheas to terminal bronchioles in comparable doses or marketed DPI formulation.	[50]
To study the effects of addition of fines and the addition sequence of fine carrier on in vitro pulmonary deposition of AmB LDPI formulations using TSI at different flow rates.	Liposome	HSPC, CHOL, α-tocopherol, SPG-3, SA	AmB	Reverse phase evaporation technique, spray freeze drying	NA	FPF (28.4%) Dispersibility index (31.8) Effective index (50.4) Addition of fines improved in vitro pulmonary deposition of LDPI AmB.	[51]
To introduce transfersomes as a carrier for pulmonary delivery in the form of DPI formulation.	Transfersome	Mannitol, lecithin, Span^®^ 60	ITZ	Thin film hydration, spray drying	NA	MMAD (5.1 ± 0.7 µm) Emission (82.7 ± 2.6%) FPF (37.3 ± 3.1%) GSD (2.1 ± 0.2) ITZ-loaded nanotransfersomes possessed suitable aerosolization performance and has the potential to be applied in the formulation of DPIs for lung delivery of various drugs.	[53]
To prepared dry powder formulation of ITZ loaded chitosan nanoparticles for pulmonary administration.	Polymeric nanoparticles	Chitosan, TPP, leucine, mannitol, lactose	ITZ	Spray drying method	NA	Sustain released of drug over 48 h. FPF achieved was around 42.9%. Addition of 10% mannitol and 10% leucine improved the aerosolization performance and pulmonary deposition.	[61]
To developed VRZ-loaded PLGA nanoparticles for pulmonary delivery.	Polymeric nanoparticle	PLGA	VRZ	Multiple-emulsification method	Swiss albino mice	Sustain released of drug over 15 days. MMAD of porous VNP was 2.29 µm while non-porous VNP was 2.58 µm. Maximum drug deposition of porous VNP was 45.55 µg/g higher than non-porous VNP.	[63]
To investigate the release profile of VNP in the lungs.	Polymeric nanoparticles	PLGA	VRZ	Multiple emulsion solvent evaporation method	Swiss albino mice and Sprague-Dawley rats	Released of VNP followed non-Fickian diffusion or anomalous diffusion. Gamma scintigraphy showed 4 h post administration of free VRZ accumulated in the kidney whereas VNP remained in the lungs.	[64]
To formulate sustained release Ch-VNP for increasing drug residence time in the lungs.	Polymeric nanoparticles	PLGA, chitosan	VRZ	Multiple emulsion solvent evaporation technique	Swiss albino mice	Sustain released of drug over 8 days. MMAD, GSD and FPF of the Ch-VNP were around 2.8 µm, 3.04 and 57.47%. AUC of Ch-VNP is 2.49 folds and 64 folds higher than the non-coated VNP and free VRZ respectively.	[65]

**Table 3:** Aerosolized intravenous formulation for nebulization against pulmonary aspergillosis.

Objective	Drug	Cell Line/Animal Model	Outcome	Source
To evaluate the effectiveness of aerosolizing the IV formulation of VCZ as prophylaxis against IPA.	VRZ	Outbred ICR mice	Inhaled aerosolized VRZ reduced the extent of invasive pulmonary aspegillosis in infected mice. Inhaled aerosolized VRZ was well tolerated by uninfected mice. The aerosolized VRZ may be effective for targeted delivery to the lungs.	[68]
To evaluate the IV solution characteristics and particle size distribution of nebulized VCZ and PCZ.	PCZ, VRZ	NA	Aerosolized VRZ MMAD (2.8 µm) FPF (76.8%) GSD (2.0) Aerosolized PCZ MMAD (3.4 µm) FPF (62.9%) GSD (2.4) The IV solutions of VRZ and PCZ showed characteristics that were suitable for aerosol delivery.	[69]
To determine by experimentation whether micafungin and anidulafungin possess physicochemical properties suitable for nebulization.	Micafungin, anidulafungin	NA	The aerosolized micafungin and anidulafungin possessed suitable physicochemical properties for proper tolerability by nebulization.	[70]

**Table 4:** Nanocarriers for lung delivery through nebulizers.

Objective	Type of Nanocarrier	Polymer Used	Drug	Method of Preparation	Cell Line/Animal Model	Outcome	Source
To prepare, characterize and evaluate performance of AmB-loaded aerosolized liposomes for their selective presentation to lungs’ alveolar macrophages.	Liposome	PC, CHOL, SA, mannan, pullulan	AmB	Thin film hydration	Albino rats of wistar origin, human RBC	Mean vesicle size (2.56 ± 0.32 µm) Aerosolized liposomal AmB coated with targeting ligand has longer retention of drug in airways than aerosolized plain drug solution. Liposomal AmB coated with targeting ligand has higher lung distribution of drug.	[71]
To compare the impact of four different commercially available nebulizers on the physicochemical properties of aerosolized liposomal AmB.	Liposome	HSPC, CHOL, DSPG	AmB	-	NA	MMAD (<2 µm) FPF < 3.3 µm (>85%) GSD (around 1.9) Administration of liposomal AmB via direct deposition into the lung may result in high concentrations in the epithelial lining fluid and airways.	[73]
To determine which doses of AmB can reach the different lung compartments when using aerosols of liposomal AmB and the possible toxic effects on the cells.	Liposome	HSPC, CHOL, DSPG, α-tocopherol	AmB	Thin film hydration	A549	Mean diameter (94 ± 0.1 nm) PI (0.110 ± 0.020) Aerosolized liposomal AmB can reach the deepest part of the lungs. The aerosol therapy with nebulized liposomal AmB could be efficient but the doses need to be carefully controlled to avoid toxicity.	[76]
To develop and characterize ITZ-loaded NLC formulation for nebulization.	NLC	Precirol ATO 5, oleic acid, Eumulgin SML 20, glycerol 85%	ITZ	Hot high pressure homogenization	NA	Particle size (around 200 nm) Burst release of 80% ITZ within 5 min from ITZ-NLC.	[77]
To evaluate the effect of autoclaving on the particle size of ITZ-loaded NLC.	NLC	Precirol ATO 5, oleic acid, Eumulgin SML 20, glycerol 85%	ITZ	Hot high pressure homogenization	NA	Particle size (100–250 nm) A unique long-term stable carrier system for poorly soluble drugs was obtained by simply autoclaving NLC.	[78]
To develop and characterize an isotonic, sterile ITZ-loaded NLC formulation for pulmonary application to treat aspergillosis in falcons.	NLC	Precirol ATO 5, super refined oleic acid, Eumulgin SML 20, glycerol 85%	ITZ	Hot high pressure homogenization	A549, falcon	Particle size (100–200 nm) No change in cell viability against A549 cells. ITZ-NLC can penetrate deeply into the respiratory tract of the falcon.	[79]
To investigate the potential of antifungal nanoemulsions for pulmonary inhalation by nebulization.	Nanoemulsion	Intralipid^®^ 20% and Clinoleic^®^ 20%	AmB	Vortex mixing, sonication	NA	AmB Intralipid^®^ VMD (5.00 ± 0.07 µm) FPF (57%) AmB Clinoleic^®^ VMD (4.41 ± 0.19 µm) FPF (80%) Lipid nanoemulsions form successful nanocarrier systems for the highly lipophilic drug AmB.	[82]
To improve the solubility and stability of AmB as a nanoparticulate colloid after reconstitution from a lyophilized dry powder with SDS.	Lipid micelle	SDS	AmB	Solvent evaporation	RBC, A549, Calu-3, Ams NR8383, ATCC 9763, *C. albicans* and *C. neoformans*	MMAD (1.74 µm) ED (±%) FPF (80%) GSD (2.0) SDS-AmB has lower toxicity against human RBC, A549, Calu-3 and Ams NR8383 cells than pure AmB.	[83]
						SDS-AmB formulation also exhibited lower MIC and MFC values against *C. albicans* and *C. neoformans* than pure AmB.	
To formulate AmB loaded depolymerised chitosan and stearic acid polymeric micelles for prevention of invasive pulmonary fungal infection.	Polymeric micelle	Chitosan, stearic acid	AmB	Solvent evaporation method	*Candida albicans, Aspergillus* *niger, Aspergillus fumigatus, Aspergillus flavus,* *Cryptococcus neoformans*	Solubility was 360 folds higher than free AmB. Nebulization efficiency around 56% and FPF ranging from 40%–50%. Stable throughout the process of nebulization.	[90]
To enhance the solubility and pulmonary delivery of ITZ by formulating ITZ loaded depolymerised chitosan and stearic acid polymeric micelles.	Polymeric micelle	Chitosan, stearic acid	ITZ	Film hydration method	*Candida albicans, Aspergillus niger,* *Aspergillus fumigatus*	Solubility was more than 1000 times higher than free ITZ. FPF between 38% to 47%. Sustain released of drug for 60 h. Stable throughout the process of nebulization.	[91]
To develop inhaled polymethacrylic acid nanoparticles containing AmB for prevention of pulmonary invasive aspergillosis.	Polymeric nanoparticles	Polymethacrylic acid	AmB	Freeze-drying method	*A. fumigatus,* lung epithelium A549 cells, monocyte-derived-macrophages, BALB/c mouse, C57BL/6 mouse	AmB-PMA had the solubility at 4 mg/mL. Immuno-suppressed infected BALB/c mice that received 3 days AmB-PMA as phophylaxis has 99% lower of fungal burden in the lungs than immuno-suppressed infected and untreated BALB/c mice. A total of 90% reduction of TNF-α in the infected BALB/c mice treated with AmB-PMA.	[92]
To develop AmB-loaded Poly (lactic acid) grafted with poly (ethylene glycol) nanoparticles for improving the adhesion and penetration of AmB into the fungi cells.	Polymeric nanoparticle	PLA, PEG	AmB	Emulsion–solvent evaporation method	Human bronchial Calu-3, alveolar (A549) cell lines, *Candida albicans, Candida parapsilosis, Candida krusei, Aspergillus fumigatus, Aspergillus* *nidulans*	Sustain released of drug over 10 days. PEG-g-PLA-AmB deposited mostly at the bronchial level then the alveolar regions. Antifungal activity of PEG-g-PLA-AmB showed 2 to 3.3 folds decrease of the half-maximal inhibitory concentration $\left({\mathrm{IC}}_{50}\right)$ value on the *Aspergillus fumigatus* compared to the free AmB.	[94]

**Table 5:** Nanocarriers for intravenous formulation against pulmonary aspergillosis.

Objective	Type of Nanocarrier	Polymer Used	Drug	Method of Preparation	Cell Line/Animal Model	Outcome	Source
To prepare ITZ-loaded liposomes coated by CMC and study the physicochemical properties, in vitro antifungal activities, safety evaluation, pharmacokinetics and tissue distribution of the liposomes.	Liposome	Soya lecithin, CHOL, CMC	ITZ	Thin film dispersion	*C. albicans*, Kunming mice	Liposomes’ diameters (349.3 ± 18 nm) ITZ-CMC liposomes showed higher mean retention time and AUC than commercially available ITZ injection. ITZ-CMC liposomes showed greater in vivo and in vitro stability. ITZ-CMC liposomes showed greater lung-targeted delivery of ITZ.	[96]
To develop an alternative liposomal formulation for AmB while using the simplest SCF-CO_2_ method and examine the effects of various factors.	Liposome	HSPC, DSPG, CHOL, DMA, ascorbic acid, lactose	AmB	Supercritical fluid of carbon dioxide method	Sprague-Dawley rats, rat RBC	Average particle size (137 nm) SCF-processed AmB achieved similar AUC as AmBisome^®^. The SCF-CO_2_ method can serve as a potential alternative for preparing liposomal AmB for industrial applications.	[97]
To prepare the LNs which can entrap poorly water-soluble drug, AmB with high drug EE in them.	SLN	DPPC, CHOL, DPPA, DSPE-mPEG_2000_	AmB	Spontaneous emulsification and solvent evaporation	293 cells, Sprague-Dawley rats, Sprague-Dawley rat RBC, *C. albicans* and *A. fumigatus*, male ICR mice	Mean particle size (84.4 ± 6 nm to 95.2 ± 2 nm) PEG-LN AmB showed higher prolonged circulation half life and higher AUC_0–24h_ than Fungizone^®^ and AmBisome^®^.	[98]
						PEG-LN AmB showed lower MIC against *C. albicans* and *A. fumigatus* than Fungizone^®^ and AmBisome^®^.	
To develop an IV formulation of ITZ using lipid nanoparticles based on binary mixture of liquid and solid lipids.	NLC	tristearin, TO, Tween 80, eggPC, DSPE-mPEG_2000_	ITZ	Melt homogenization	Male Sprague-Dawley rats	Particle size (192.7 ± 14.4 nm to 241.9 ± 13.5 nm) ITZ-NLC showed possible controlled release property. ITZ-NLC showed sustained release property with less liquid lipid proportion.	[99]
To develop high payload ITZ-incorporated lipid nanoparticles with modulated release property using a binary mixture core of solid and liquid lipid for oral and parenteral administration.	NLC	tristearin, TO, Tween 80, eggPC, DSPE-mPEG_2000_	ITZ	Hot high-pressure homogenization method	Male Sprague-Dawley rats	Particle size (285.9 ± 14.8 nm) PI (0.195 ± 0.015) High payload ITZ-NLC showed controlled release property. High payload ITZ-NLC showed initial burst release for 4 h followed by sustained release for 24 h.	[100]
To develop a lung-specific delivery system of AmB with a high pulmonary distribution and a low nephrotoxicity.	Lipid micelle	DCH	AmB	Vortex mixing	Oncins France 1 male mice	Particle size (404.9 ± 1.7 nm) AmB-DCH has higher drug delivery to lung and 15-fold lower drug concentration in kidney as compared to liposomal AmB formulation.	[101]
To develop AmB loaded D-α-tocopheryl polyethylene glycol 1000 succinate-b-poly (ε-caprolactone-ran- glycolide) nanoparticles and evaluate its in vitro and in vivo antifungal activity.	Polymeric nanoparticles	PLGA, TPGS 1000	AmB	Modified nanoprecipitation method	*C. albicans,* BALB/c mice	Sustain release of drug over 18 days. AmB-NP showed approximately 50% lower of log CFU/gram of *C. albicans* in the infected mice than the free AmB.	[102]
						AmB-NP showed 50% higher of survival rate in the infected mice than the free AmB.	
To evaluate the fungistatic and fungicidal effects of AmB loaded D-α-tocopheryl polyethylene glycol 1000 succinate-b-poly (ε-caprolactone-ran- glycolide) nanoparticles for treatment of pulmonary fungal infection.	Polymeric nanoparticles	PLGA, TPGS 1000	AmB	Double emulsion method	*C. glabrata,* BALB/c mice	Sustained released of drug for 22 days. PLGA-AmB and AmB-NP had the similar MIC value. AMB-NP had high affinity to the fungi cells. No detectable lesions and oedema found and lower fungal burden after treated with AmB-NP. AmB-NP showed 40% higher of survival rate in the infected mice than the PLGA-AmB.	[104]

## Abbreviations

DPPA	1,2-Dipalmitoyl-*sn*-glycero-3-phophate (monosodium salt)
DPPC	1,2-Dipalmitoyl-*sn*-glycero-3-phosphocholine
DSPE-mPEG_2000_	1,2-Distearoyl-*sn*-glycero-3-phosphoethanolamine-N-[methoxy(polyethylene glycol)-2000] (ammonium slat)
AIDS	Acquired immunodeficiency syndrome
ABPA	Allergic bronchopulmonary aspergillosis
IPA	Invasive pulmonary aspergillosis
CPA	Chronic pulmonary aspergillosis
AmB-NP	AmB-loaded PLGA-TPGS nanoparticles
Nano-D-AmB	AmB-loaded with PLGA and DMSA polymeric nanoparticles
AmB-DCH	AmB prepared with sodium deoxycholate
AmB	amphotericin B
PEG-g-PLA-AmB	AmB-loaded polymeric nanoparticles with PLA-grafted with PEG
CO_2_	Carbon dioxide
CMC	Carboxymethylchitosan
Ch-VNP	Chitosan-coated PLGA nanoparticles containing VRZ
CCEs	Cholesteryl carbonate esters
DCSA-AmB	Depolymerised chitosan and stearic acid
DMSA	Dimercaptosuccinic acid
DSPG	Distearoylphosphatidylglycerol
DPI	Dry powder inhaler
ED	Emitted dose
EE	Entrapment efficiency
FPF	Fine particle fraction
GSD	Geometric standard deviation
HIV	Human immunodeficiency virus
HSPC	Hydrogenated soy phosphatidylcholine
CHOL	Cholesterol
ITZ	Itraconazole
LDPI	Liposomal DPI
VLPP	Microparticulate dry powder formulations containing voriconazole
MIC	Minimum inhibit concentration
MFC	Minimum fungicidal concentration
PA	Pulmonary aspergillosis
Nano-D-ITZ	PLGA-DMSA nanoparticles containing ITZ
PLGA	Poly-lactide-co-glycolide
AmB-PMA	Polymethacrylic acid nanoparticles containing amb
PCZ	Posaconazole
SCC	Sodium cholesteryl carbonate
SDS	Sodium deoxycholate sulfate
SD	Solid dispersions
SPG-3	Soy phosphatidyl glycerol
SCF	Supercritical fluid
TA	Tartaric acid
SA	Succinic acid
FA	Fumaric acid
MA	Malic acid
TFF	Thin film freezing
TPP	Tripolyphosphate
TB	Tuberculosis
TNF-α	Tumour necrosis factor
VRZ	Voriconazole
VNP	Voriconazole-loaded PLGA nanoparticles
